# Modulating C_**2**_ Selectivity in CO_**2**_ Electroreduction through Molecular Surface Engineering of Copper Nanowires

**DOI:** 10.1021/acsaem.5c02727

**Published:** 2025-11-11

**Authors:** Andrea Conte, Chiara Alberoni, Silvia Carlotto, Marco Baron, Sara Bonacchi, Alessandro Aliprandi, Sabrina Antonello

**Affiliations:** Department of Chemical Sciences, 9308 University of Padova, Via F. Marzolo 1, Padova 35131, Italy

**Keywords:** Cu nanowires, electrocatalysis, ethylene, CO_2_ reduction, surface engineering

## Abstract

Electrocatalytic reduction of CO_2_ to multicarbon products represents a key target in the development of artificial (photo)­synthetic systems. Copper-based electrodes are uniquely suited for this purpose, yet achieving high selectivity toward C–C coupled products such as ethylene, over undesired byproducts like methane and hydrogen, remains a major challenge. Surface engineering has emerged as a powerful strategy to steer the selectivity of CO_2_ electroreduction toward ethylene. Here, we introduce a modular hybrid electrode architecture composed of copper nanowires (CuNWs) coated with a thin, functional organic shell formed via electroreduction of ethylene-bridged dipyridophenazine (*dppz*) dication derivatives. This core–shell architecture enables fine-tuning of the interfacial catalytic environment through rational molecular design. We demonstrate that subtle structural variations affecting the electronic distribution of the phenazine moiety have a profound effect on ethylene selectivity. Notably, electrodes incorporating electron-withdrawing groups achieved nearly a tenfold increase in Faradaic efficiency for ethylene relative to pristine CuNWs, whereas hydrophilic functionalities favored hydrogen production and suppressed C_1_ and C_2_ products. DFT calculations reveal how the substituents alter local electric fields and interfacial water binding, providing a molecular-level rationale for the observed trends. Ex situ characterization of core–shell electrodes further reveals that the polymeric coating stabilizes the Cu surface against corrosion and provides valuable insights into the structural reconstruction of CuNWs during the electrocatalytic process. This work not only advances the fundamental understanding of hybrid interface effects by providing a powerful and scalable approach for decoupling catalyst selectivity from the intrinsic properties of the metal surface but also offers a promising route toward efficient and industrially relevant carbon conversion technologies.

## Graphical abstract

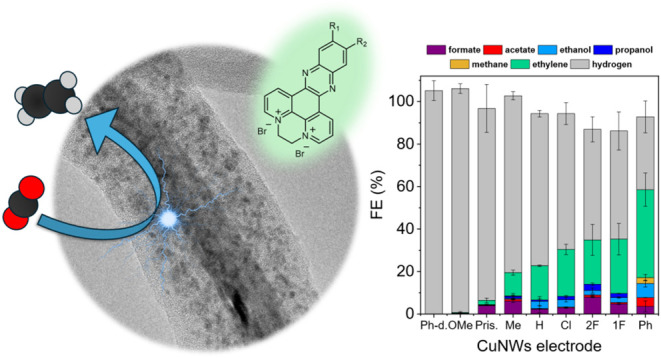

## 1 Introduction

The urgent need to combat climate change and achieve energy independence has driven intense interest in developing sustainable technologies aimed at reducing greenhouse gas emissions and transitioning toward a carbon-neutral economy.^1–3^ Among these, electrochemical CO_2_ reduction has emerged as a promising strategy. This approach enables the conversion of CO_2_, a major contributor to global warming, into value-added chemical products, thereby mitigating its environmental impact while simultaneously supporting renewable energy systems.^4–8^ Of the various possible CO_2_ reduction products, ethylene stands out due to its broad industrial relevance, spanning from plastics manufacturing to applications in renewable energy storage and conversion.^9–17^ However, the electrochemical conversion of CO_2_ to ethylene remains highly challenging since the reaction involves complex multielectron pathways; therefore, achieving high selectivity and efficiency in ethylene production, despite other C_1_ or C_2_ products such as ethanol,^18^ acetate,^19^ formate,^20^ and methane,^6^ requires fine control over the catalyst’s activity, surface chemistry, and reaction kinetics.

Surface engineering of electrodes has emerged as a powerful strategy to overcome these challenges, as it allows precise control over both the catalyst’s crystal surface and its electronic structure at the molecular level.^21^ In this framework, Cu nanowires (CuNWs)-based catalysts are particularly appealing not only for the earth abundance of copper, its low cost, and unique ability to catalyze CO_2_ reduction to hydrocarbons^6,22–25^ but also, thanks to their high surface area-to-volume ratio, their inherent readiness to form self-standing porous conductive networks, the presence of (100) structures, which have been predicted to be beneficial for C_2_ formation,^26^ and the improved mass transport of both reactants and products.^27–29^ From a molecular point of view, recent studies have demonstrated that N-heterocyclic salts represent effective molecular dopants for tuning the surface properties of Cu electrodes.^21,30–43^ By forming coordination complexes or charged oligomeric layers, the electron-rich nitrogen center within the N-heterocyclic compounds such as pyridine,^44^ pyrrole,^45^ imidazole,^46^ viologen,^47^ carbenes,^48^ and phenanthrolinium salt^49^ substantially alters the Cu surface chemistry by charge transfer processes, thus influencing the overall adsorption energies of CO_2_ as well as the key intermediates.^48^

In this work, we focus on the potential synergistic effects arising from the interplay between metal nanostructuring, particularly the enhancement of (100) crystallographic surface facets on CuNWs, and the molecular-level engineering of the solid–liquid–gas interface to tune hydrophobicity, charge distribution, and local electric-field effects. Through this dual design strategy, we systematically modulate the local reaction environment and the intermediate adsorption behavior by design, thereby steering the catalytic process toward improved selectivity for C_2_ products, in particular ethylene. We demonstrate that the formation of an organic shell made of novel N-heterocyclic compounds onto the surface of the CuNWs core offers a promising route for molecular-level control over the CO_2_ reduction process, leveraging the unique catalytic properties of specific (100) surface structures and the dynamic interface role in stabilizing key reaction intermediates. Overall, this work aims to contribute to the establishment of design principles linking molecular dipole moments to catalytic selectivity, offering a predictive and scalable framework for selective CO_2_ conversion.

## 2 Experimental Section

### 2.1 Reagents

All manipulations were carried out in air by using standard laboratory equipment. All of the reagents and solvents were obtained from commercial suppliers and used as received without further purification. Copper dichloride dihydrated (CuCl_2_·2H_2_O, >99.0% from Merck), 1-octadecyl amine (ODA, from Merck), glucose (>99.5%, from Merck), potassium bicarbonate (>99.7%, from Merck), ethanol (>99.5%, from Merck), and isopropanol (>99.8%, from Merck) were used. The precursors for the synthesis of organic additives were provided by Merck, Alfa-Aesar, TCI Chemicals, and BLDpharm.

### 2.2 Preparation of CuNWs

The CuNWs syntheses were performed in a 500 mL flask, closed by a screw cap. A thermostated oil bath was employed to heat the reaction mixture. Typically, 0.400 g of CuCl_2_·2H_2_O and 2.529 g of octadecylamine (ODA) were added to 200 mL of distilled water. The mixture was mixed and sonicated for 40 min at 40 °C. Afterward, 1 equivalent of glucose with respect to CuCl_2_·2H_2_O was added to the reaction flask, mixed, and heated for 18 h at 125 °C without stirring the solution. The reaction flask was cooled to room temperature. A red-brownish slurry was formed. Dark red CuNWs precipitated and accumulated at the bottom of the reaction flask. The product was centrifuged for 5 minutes using various aliquots of deionized water. Further centrifugations were carried out using isopropanol. The obtained CuNWs were stored at 20 °C in isopropanol under an argon-saturated atmosphere.

### 2.3 CuNWs-Based Electrode Preparation

CuNWs dispersed in isopropanol were deposited on a nonconductive borosilicate glass slide, which served only as mechanical support for the catalyst film, and heated at 150 °C (Figure S1B). The electrode holder (used to immerse the CuNWs electrode in solution) was clamped with an alligator clip in direct contact with the CuNWs. Once immersed, the percolation network was generated under an applied potential of −1.3 V vs RHE for 60 s (Figure S2). After deposition and activation, the nanowire network itself provided sufficient electrical conductivity for electrochemical measurements. A resistance value of approximately 0.2 Ω was estimated after the activation step using a two-point probe measurement taken at the opposite edge of the 3 cm-long glass slide.

### 2.4 Electrodeposition of Organic Additives on CuNWs Electrodes

The electrodeposition of the organic additives on the CuNWs surface was standardized as follows: two cyclic voltammetry scans from +0.45 V to −1.3 V vs RHE, followed by the application of a fixed potential of −0.6 V vs RHE for 800 s.

### 2.5 Materials Characterization

#### 2.5.1 Scanning Electron Microscopy (SEM)

Images were collected with a Zeiss Sigma HD microscope equipped with a Schottky FEG source, one detector for backscattered electrons, and two detectors for secondary electrons (InLens and Everhart Thornley). The microscope is coupled to an EDX detector (from Oxford Instruments, X-act PentaFET Precision) for X-ray microanalysis, working in energy-dispersive mode. A low accelerating voltage (5 kV) was used to accurately delineate fine surface features and to avoid conductive coatings that can deleteriously modify the surface morphology.

#### 2.5.2 High-Resolution Transmission Electron Microscopy (HR-TEM)

Measurements were carried out using a JEOL JEM-F200 S/TEM working at 200 kV and equipped with a Cold Field Emission Gun.

#### 2.5.3 Mass Spectrometry

Agilent 6550 iFunnel Q-TOF LC/MS System with UPLC 1290 Infinity (Agilent, CA, United States) was employed to carry out the MS analysis.

#### 2.5.4 Raman Spectroscopy

The Renishaw inVia micro-Raman, with laser sources at 633 nm, was used for all Raman experiments.

#### 2.5.5 X-ray Diffraction

Film XRD measurements were conducted using a Bruker D8 Advance diffractometer, fitted with a LYNXEYE detector in 1D mode. Diffraction data were acquired by exposing powder samples to CuKa1,2 X-ray radiation. X-rays were generated from a Cu anode supplied with 40 kV and a current of 40 mA. The data were collected over the 30–1001 2y range with a step size of 0.0251 2y and a nominal time per step of 0.20 s. Fixed divergence slits of 0.501 were used together with Soller slits with an aperture of 2.341.

#### 2.5.6 Electrochemical Characterization of the CuNWs Surface

We performed cyclic voltammetry in a 1 M NaOH solution in a three-electrode cell for the pristine CuNWs electrode. The reference electrode was Hg/HgO (1 M NaOH), and the counter electrode was a copper plate. The electrochemical measurements were performed by using a Biologic P300 potentiostat.

### 2.6 Electrocatalytic Tests

Constant potential electrolysis (CPE) (1.5 h) was performed in 1 M KHCO_3_ (Merck, 99.7%) using an H-type cell separated by a proton-exchange membrane (Nafion 117). The cathodic compartment contained 20 mL of electrolyte (1 M KHCO_3_) and housed the working electrode (exposed geometric surface area: 12 mm × 20 mm (2.4 cm^2^) and the reference electrode (saturated calomelan electrode (SCE)), while the anodic compartment contained 20 mL of electrolyte and a graphite bar as the counter electrode. The electrolyte was purged with CO_2_ gas (99.999%, Air Liquide) also during the entire electrolysis time. The solution in the cathodic compartment was stirred during the electrolysis using a magnetic stirrer. The electrochemical measurements were performed using a CHI Instrument 660C potentiostat. The iR drop was compensated during the experiments. The gas was collected using a sampling bag (Supelco), and the gas was analyzed by means of a gas chromatograph (GC, Agilent 8860A) for detection of gas products. Liquid products were analyzed after the electrolysis experiments. A Bruker Avance 300 MHz (7.05 T) operating at 300.1 MHz was employed. NMR tubes were prepared by adding 100 μL of D_2_O, and 10 μL of 8 mM solution of DMSO in D_2_O with 500 μL of cathodic solution. The FE of each product was calculated by the following equation after quantification:

1 FE=nZF/Q

where *n* represented the number of moles of the product, *Z* was the number of electrons exchanged per CO_2_; *F* is the Faraday constant (96485 C/mol), and *Q* is the amount of charge that passes through the working electrode. All the reported potential values are referenced to RHE.

### 2.7 Electrochemical Impedance Spectroscopy Experiments

The electrochemical studies were carried out using a CHI660c instrument potentiostat and CHI660c software. The experiments were done in a three-electrode configuration, which consisted of a saturated calomel electrode (SCE) as the reference, a graphite bar as the counter, and a CuNWs electrode as the working electrode. The electrochemical impedance spectroscopy (EIS) measurements were made in 1 M KHCO_3_, at different potentials, using a perturbation of 5 mV for frequencies ranging from 100 kHz to 1 Hz at 10 steps/decade. The impedance spectra were analyzed by fitting them to an equivalent electrical circuit using NOVA 2.1.

### 2.8 Computational Details

All quantum mechanical calculations were carried out by using the ORCA program (version 5.0.0).^50^ All structures were optimized using the hybrid B3LYP functional.^51^ The def2 basis set (def2-TZVP) of the Karlsruhe group was used. def2-TZVP is a valence triple-zeta basis set with “new” polarization functions. Dispersion corrections were included by adopting Grimme’s DFT-D3 approach with D3BJ, an atom-pairwise dispersion correction to the DFT energy with Becke–Johnson damping.^52^

## 3 Results and Discussion

### 3.1 Cu Nanowires-Based Electrode Preparation and Electrocatalytic Tests

CuNWs were synthesized according to a previously reported protocol.^29^ Analysis of the scanning electron microscopy (SEM, Figure S1A) images revealed an average diameter of (75 ± 15) nm and a high aspect ratio. A suspension in isopropanol of the so-obtained CuNWs was drop-cast onto preheated borosilicate glass slides (150 °C) to fabricate our electrodes, as schematically illustrated in Figure S1B. To activate charge transport within the CuNWs network, a reductive potential of −1.3 V vs RHE was applied to remove the intrinsic insulating behavior caused by the presence of the surface-bound capping agent (octadecylamine) and the native CuO layer formed on the NW surface during synthesis. A drastic increase in current was observed after polarizing the electrode for approximately 10 s (see Figure S2). This procedure allowed us to exploit our CuNWs as active catalytic material without interference or bias effects from external conductive supports, thus enabling us to get insights into their intrinsic electrocatalytic properties.^53^ Furthermore, the strong and spontaneous adhesion of CuNWs to the glass substrate eliminates the need for polymeric binders (e.g., Nafion), simplifying the electrode preparation process. Cyclic voltammetry (CV) in a 1 M NaOH electrolyte was performed to correlate the well-known hydroxide ion adsorption and desorption electrochemical peaks to the exposed crystallographic facet structures of Cu surfaces. In particular, a sharp peak at +0.37 V vs RHE was assigned to OH^–^ electro-oxidation to oxygen adsorbate species on (100)-rich defect facets, while a second peak at +0.41 V vs RHE was referred to (110). A broad peak around −0.10 to −0.05 V vs RHE was attributed to OH^–^ adsorption on large (100) terrace domains, confirming that the CuNWs are predominantly terminated by (100) facets, consistent with the well-known penta-twinned structure of the CuNWs (Figure S3).^54–56^ To evaluate the electrocatalytic performance of our CuNW-based electrodes, CO_2_ electroreduction experiments were carried out in a two-compartment H-type cell using 1 M KHCO_3_ as the electrolyte and keeping a fixed reductive potential for 1.5 h. The pristine CuNWs (**CuNWs@Pristine**) electrode was first tested at −1.0 V vs RHE, selected as the reference potential for Cu-based catalysts; hydrogen was detected as the dominant product with a Faradaic efficiency (FE) of ∼90%. Only approximately 3% of ethylene and 4% of formate with minor traces of ethanol and CO were quantified.

To suppress the competing H_2_ evolution reaction (HER) and systematically investigate the influence of electronic properties of a molecular layer on the catalytic behavior of CuNWs, we designed, for the first time, a library of phenanthrolinium salt derivatives (Figure 1). They were inspired by *N*,*N*′-ethylene-phenanthrolinium dibromide (**Ph)** that represents the benchmark in this field with the best selectivity toward C_2_ products when bulk Cu is used as the electrode.^31,33^ In particular, in the series of ethylene-bridged bipyridyldiylium-phenazine (*dppz*) derivatives, different substituents, referred to as **2F**, **1F**, **Cl**, **H**, **Me**, **OMe**, were synthesized to be used as organic additives for coating the CuNW’s surface. Furthermore, we also synthesized and tested **Ph-diket** to expand our knowledge of the effect of hydrophilic functionalities.

**Figure 1:**
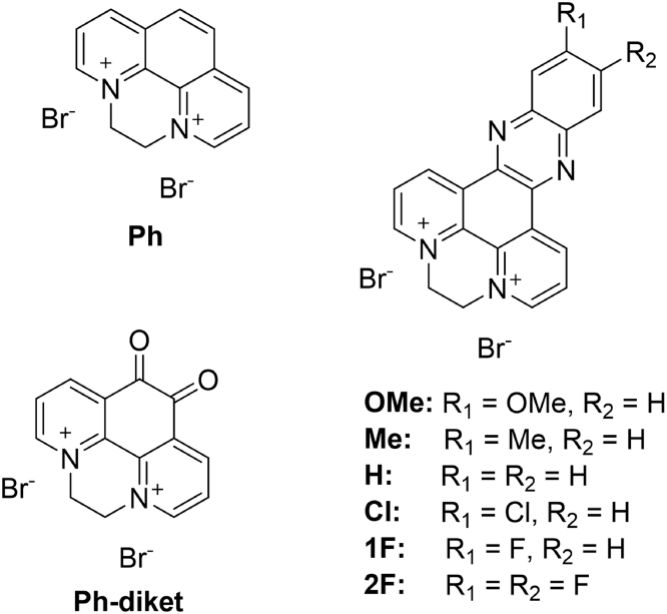
Schematic representation of the organic dicationic salts employed in this work as additives for the electromodification of CuNWs. For clarity, the abbreviations used throughout the manuscript are indicated above each molecular structure.

The dicationic molecules were deposited onto the electrode surface by electroreduction under both potentiodynamic and potentiostatic conditions. In detail, the additive (1 mM) was dissolved in the cathodic compartment of an H-cell together with the CuNWs electrode, and CV was performed over a potential window ranging from +0.45 V to −1.3 V vs RHE (Figure 2A shows as an example the **CuNWs@Ph** behavior). Distinct Faradaic activity was observed between +0.25 V and −0.4 V and was assigned to the onset of the electropolymerization onto the CuNWs surface of the organic monomer in solution. Interestingly, the subsequent scan showed a consistent reduction in the redox activity, suggesting that a single voltammetric sweep passivates the nanowire. An analogous procedure was followed for all *dppz* derivatives, which exhibited essentially the same voltammetric behavior and features as **Ph**, as exemplified by **1F** in Figure S4A. SEM and energy-dispersive X-ray spectroscopy (EDX) images (Figures 2B–D and S5–S6 and Table S1) confirm that, following the first reductive potential scan, the CuNWs surface becomes encapsulated with a uniform carbonaceous layer (shell) with an average thickness of 17 nm. STEM–EDS mapping (Figures S7–S8) allowed us to confirm also the presence of N atoms in the layer. To assess the evolution of this organic shell, SEM analysis was also carried out after chronoamperometry at −0.6 V vs RHE for 800 s (Figure S4B), revealing a polymer thickness increase to approximately 30 nm (Figure 2C), while a constant average diameter of the CuNWs metal core was detected (75 ± 20 nm). Raman spectroscopy carried out on pristine CuNWs and CuNWs modified with the organic shell (Figure S9) revealed the presence of characteristic vibrational bands consistent with the phenanthrolinium derivative in the modified samples, whereas these bands were absent in pristine CuNWs. The pristine CuNWs indeed display a broad band around 520–550 cm^–1^, corresponding to Cu–O vibrations from a thin surface oxide layer, which includes Cu_2_O and CuO. Upon modification, new well-defined peaks appear in the 1400–1650 cm^–1^ region, attributable to the C=C and C=N stretching vibrations of the aromatic and heterocyclic rings of the ligands. In particular, the **CuNWs@Ph** and **CuNWs@diket** samples exhibit peaks at ∼1470 and ∼1590 cm^–1^, characteristic of 1,10-phenanthroline, while the **CuNWs@1F** and **CuNWs@2F** samples show additional features near ∼1340, ∼1480, and ∼1600 cm^–1^, consistent with di-pyridyl-phenazine presence.^57,58^ These spectral features confirm the presence and chemical identity of the organic coating on the CuNWs. Whereas ESI-TOF-MS analysis of the solution used during the CuNWs electrodeposition indicates the formation of oligomers composed predominantly of two repeating units (dimers), yet species of higher molecular weight were also detected, suggesting that the electrodeposited shells consist of a mixture of oligomers with varying chain lengths (Figure S10 and Table S2). Tentative structures of the trimers are shown as an example in Figure S11. This complementary evidence further substantiates the presence and composition of the organic coating. Notably, the CV profile of **CuNWs@Ph** shows decreased activity toward H_2_ generation, with a delayed onset, as shown in Figure 2.

**Figure 2:**
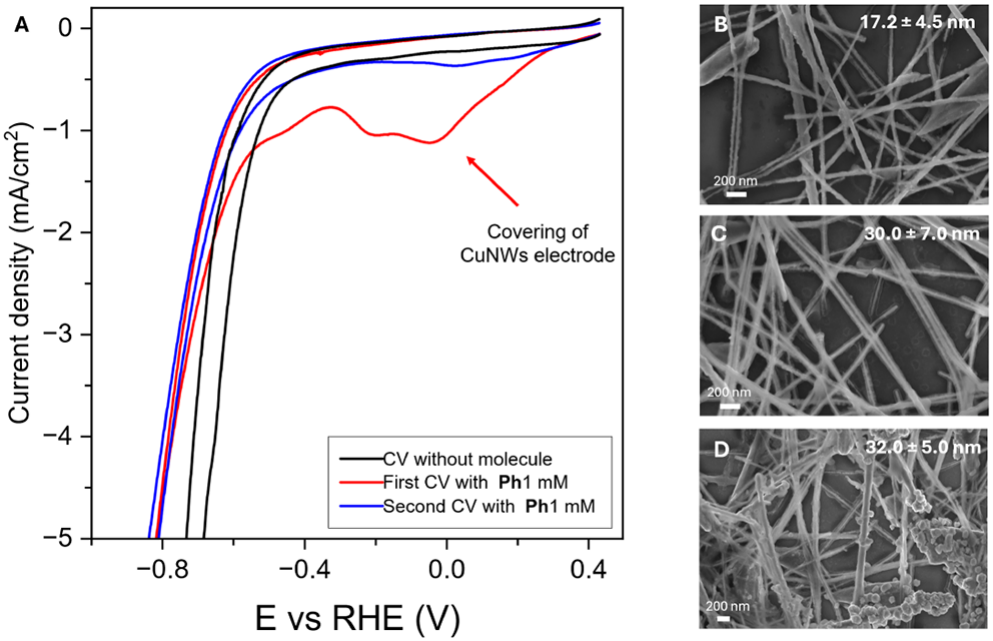
A) Cyclic voltammetry of CuNWs in 1 M KHCO_3_, in different conditions explained in the legend. B) SEM images of the CuNWs electrode after the first CV performed in the presence of 1 mM **Ph**. C) SEM image of the CuNWs electrode after the first CV and 800 s activation at −0.6 V vs RHE with 1 mM **Ph**; D) SEM image of the CuNWs electrode after the catalytic test. The average thickness of the organic coating layers is reported in each image.

CVs in alkali conditions were performed after the organic shell formation, confirming the presence of the OH^–^ adsorption peaks typical of the (100) orientation but featuring a lower intensity compared to the pristine electrode due to surface coverage by the organic shell, which limits the access of hydroxide yet guarantees the permeability to solubilized species (Figure S12). However, the shape of the peaks ensures that the electrode is not affected by significant internal ohmic drops and that the percolative contact points between CuNWs remain largely unchanged after the growth of the shell.

Figure 3A–C summarizes the key outcomes of our electrocatalytic experiments performed at −1.0 V vs RHE for 1.5 h (representative chronoamperometric curves are shown in Figure S13). In particular, the electronic effects of the substituents on the dipyridophenazine scaffold appear to play a significant role in influencing the reaction selectivity, as evidenced by the FE for ethylene production, which increases with the electron-withdrawing strength of the substituent on the phenazine moiety, while the HER progressively decreases (Figure 3A). Based on FE for ethylene, a clear trend in catalytic efficiency can be indicated following the **Ph** > **1F** > **2F** > **Cl** > **H** > **Me** > **OMe** ≈ **Ph-diket** order (Figure 3A), while the sum of C_2_ products followed the trend **Ph** > **1F** ≈ **2F** ≈ **Cl** > **H** > **Me** > **Ph-diket** (Figure 3C). A preliminary conclusion that can be drawn from these data is that the spatial confinement of CO_2_ within the shell alone is not sufficient to steer selectivity, whereas the molecular design plays a crucial role.

**Figure 3:**
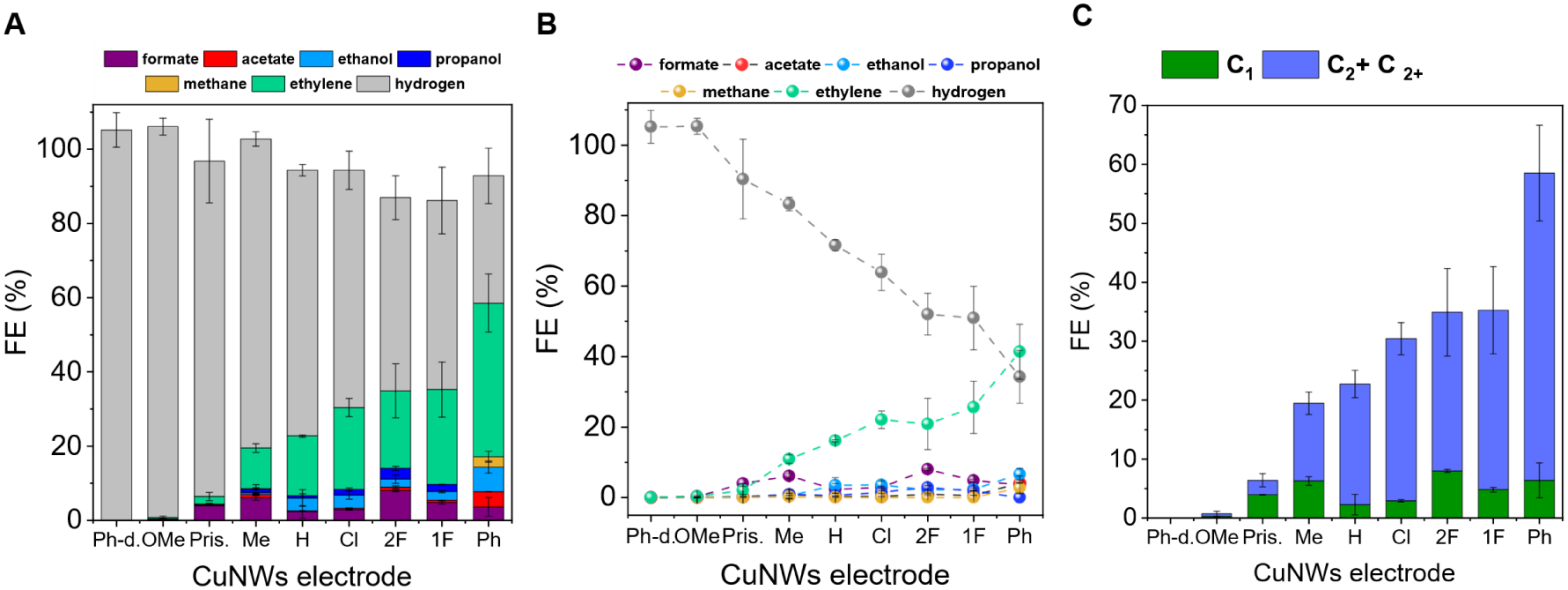
A) FE of CO_2_ reduction products obtained at −1.0 V vs RHE using **CuNWs@X** electrodes (**X** = **Ph**, **1F**, **2F**, **Cl**, **H**, **Me**, **Pristine (Pris.)**, **OMe**, **Ph-diket (Ph-d.)**), each modified with 1 mM of the respective compound in 1 M KHCO_3_, represented as bar charts. B) Corresponding spike line representation of the FE for different **CuNWs@X** electrodes. C) C_1_ and C_2+_ product selectivity for the same series of **CuNWs@X** electrodes at −1.0 V vs RHE.

In terms of total current density at −1.0 V vs RHE, the electrodes modified **CuNWs@X** with **X** = **Ph**, **2F**, **1F**, **Cl**, **H**, and **Me** exhibited values ranging from −8 to −15 mA cm^–2^ (Figure S14), only slightly lower than that of the pristine electrode, which showed an average current density of −16 mA cm^–2^. Therefore, no evident effects of the mass-transfer limitation of CO_2_ toward the electrode due to the presence of the organic layer were observed. A higher current value was observed for **CuNWs@OMe**, which is attributed to its efficient H_2_ production. Stability tests conducted over 3 h of continuous electrolysis demonstrated that the catalysts maintained consistent electrochemical behavior throughout the operation (Figure S15). Notably, **CuNWs@2F** exhibited a slight increase in relative ethylene production over time, a trend not observed for **CuNWs@Ph.** A particularly high ethylene-to-ethanol FE ratio was also observed for the fluorinated *dppz* derivatives (Table S3), while **CuNWs@Ph** displayed an FE ratio of 8.0, significantly higher than that reported when the same **Ph** molecule was used to coat polycrystalline Cu electrodes.^31^ Importantly, this value is even higher than other electrocatalytic systems reported in the literature so far, as highlighted in our comparison study in Table S4. This enhanced selectivity addressed a long-standing challenge in the field and was ascribed to the synergistic effects of the surface-bound oligomeric coating together with the prevalence of exposed Cu(100) facets on the nanowire surface. This environmental arrangement affects post C–C coupling steps, favoring deoxygenation to ethylene while disfavoring the additional proton/electron transfers needed to produce ethanol.^59^ Furthermore, the interfacial water-proton activity could play a role. Indeed, the organic layer on high-curvature CuNWs creates a more hydrophobic, lower-water-activity interface and elevates the local pH. While Cu(100) still promotes CO–CO coupling (*OCCO/*OCCOH formation), the reduced availability of interfacial protons selectively suppresses the subsequent hydrogenation steps that convert C_2_ oxygenates toward ethanol. Ethylene, which is favored under lower proton activity, becomes the dominant C_2_ product.^60,61^

Noteworthy, lowering the electrolyte concentration from 1.0 to 0.1 M KHCO_3_ shifts the product distribution toward higher fractions of ethanol and formate, with a concomitant decrease in ethylene at comparable potentials. We attribute this trend to the reduced ionic strength and buffer capacity at 0.1 M, which modify the interfacial pH and K^+^ activity. Alkali metal cations are known to modulate the local interfacial environment by affecting the electrode charge density, the electric field across the double layer, and the distribution of water molecules near the electrode.^62,63^ A larger concentration of K^+^, by increasing the interfacial electric field, can better stabilize key polar adsorbate intermediates such as *CO and lower the barrier for C–C coupling relative to the protonation pathways. The qualitative increase in ethanol and formate selectivity observed in Table S2 is therefore consistent with electrolyte-dependent behavior in the CO_2_RR on Cu.

The partial current density for H_2_ generation was, in general, reduced compared to **CuNWs@pristine**, as shown in Figure S14, except for the **CuNWs@OMe** electrode, that exhibits a higher H_2_ partial current density, reaching a value of −20 mA/cm^2^. To gain insight into the large difference in H_2_ production between pristine and modified CuNWs-based electrodes, a comparative investigation of the heterogeneous electron transfer kinetics of **CuNWs@Pristine**, **CuNWs@Ph**, **CuNWs@OMe**, and **CuNWs@Ph-diket** was conducted using electrochemical impedance spectroscopy (EIS) (Figure S16). Measurements were carried out over a frequency range of 100 kHz to 1 Hz at −0.6 V vs RHE. While all three electrodes exhibited similar qualitative features in their impedance responses, the magnitude varied significantly. The Nyquist plots were fitted using the equivalent circuit models shown in Figure S17, highlighting that the electrodes more active toward HER, **CuNWs@OMe**, **CuNWs@Ph-diket**, and **CuNWs@Pristine** showed similar impedance responses, with slightly lower resistance for the **OMe**-modified electrode, while the **CuNWs@Ph** electrode displayed a larger semicircle, referring to a higher charge-transfer resistance in agreement with a suppressed HER. EIS data suggest that the hydrophilic shell enhances ionic access and charge transfer, the pristine surface exhibits intermediate behavior, and the hydrophobic shell introduces significant barriers to both charge and mass transport. This electrochemical picture matches the expected wetting and solvation physics at the solution/organic/oxide/CuNWs junction.

The effect of the applied potential on the electrocatalytic performance was tested on **CuNWs@Ph**. Figure 4A shows that at −1.0 V vs RHE, the combined FE for C_2_ and C_2+_ products reached approximately 50%, significantly surpassing the C_1_ products and aligning with the performance of state-of-the-art catalysts (see Table S4 and Figure 3C);^31^ moreover, an important efficiency of 41% for ethylene production was achieved, with a corresponding total current density of approximately −10.1 mA/cm^2^. A further increase in the applied potential leads to a decline in formate production but an increase in the FE for ethanol, indicating a shift in the product distribution toward more reduced C_2_ species (Figure 3B). As expected, the total current density increased with a more negative applied potential (Figure S18). Between −1.0 V and −1.2 V vs RHE, the FE for ethylene remained relatively stable, allowing us to achieve a partial current density of −5.5 mA/cm^2^ for ethylene when operating at −1.2 V vs RHE. In parallel, the partial current density for ethanol also increased with more negative potentials, whereas that of formate decreased to −0.36 mA/cm^2^, indicating effective suppression of the formate production pathway. The cumulative FE of the C_1_ products (Figure 4C) decreased with decreasing applied potential, consistent with a progressive increase in C_2_ and C_2+_ species. Traces of n-propanol were also detected at −1.1 and −1.2 V vs RHE, highlighting the onset of C_3_ product formation.^6,64^

**Figure 4:**
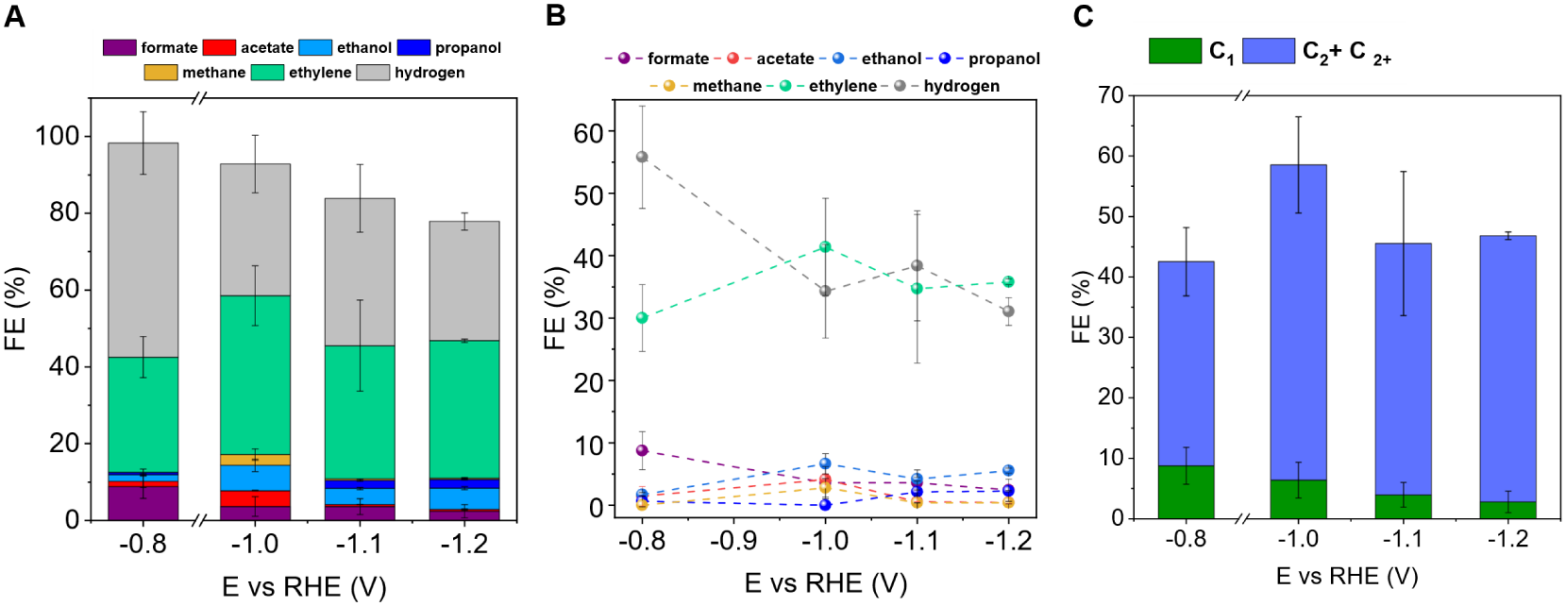
A) Faradaic efficiencies of CO_2_ reduction products obtained using **CuNWs@Ph** electrodes at various applied potentials, with 1 mM **Ph** in 1 M KHCO_3_, represented as bar charts. B) Corresponding spike line graph of the FE trends from panel A. C) Comparison of cumulative FE for C_1_ and C_2+_ products under the same conditions.

Overall, our electrocatalytic performance, yet in agreement with recent literature on polycrystalline Cu electrodes,^33,49^ possesses a key important advantage, thanks to the tenfold lower concentration of the phenanthrolinium precursor (**Ph**) employed (1 mM vs 10 mM),^49^ while keeping high selectivity and efficiency for ethylene production.

To demonstrate that the organic shell acted solely as a surface modifier without contributing to the total amount of carbon atoms in the reaction product, a control test was performed under identical conditions but in the absence of CO_2_, that is by applying a potential of −1.0 V vs RHE in Ar-saturated 1 M KHCO_3_. The blank experiment produced only H_2_, thus indicating that the passivating organic shell is not the source of carbonaceous material attributed to CO_2_ reduction (see Table S2). Moreover, postcatalysis SEM analysis revealed that the thickness of the organic shell remained largely unchanged after CO_2_ electrocatalysis, with an estimated thickness of approximately 32 nm (Figures 2D), comparable to the initial conditions. This insight demonstrates that the different selectivity toward ethylene of our **CuNWs@X** (Figure S19) is not ascribable to changes in the shell thickness.

Further investigation was also carried out to analyze the role of our organic shell on the CuNWs core’s stability. After 1.5 h of CO_2_RR electrolysis, important corrosion and reconstruction features were observed in the **CuNWs@Pristine,** such as a reduction of 24% of the CuNWs’ diameter, while only a decrease of only 7% was observed when **CuNWs@X** was used as an electrode under the same conditions. By ex situ high-resolution transmission electron microscopy (HR-TEM), we compared the morphology of our **CuNWs@X** before and after the electrocatalytic test performed both in the presence and in the absence of the passivating molecule in solution. Figure 5C–E shows metallic nanostructures embedded within the organic shell, which likely originate from the partial corrosion of the CuNW core during the electrocatalytic process. A thinner and nonuniform oligomer shell was also observed when no passivating molecule was present in the solution during the electrocatalysis, as shown for **CuNWs@1F** in Figures 5E and S20–S21. Importantly, the damaged shell adversely affects the catalytic performance of our electrodes, as evidenced by the reduced ethylene production, alongside a boost in H_2_ efficiency (Figure S22). These results point out that the presence of monomers in solution plays a critical role in replenishing or repairing the organic layer during electrocatalysis and in retaining hydrophobic environments that appear to be fundamental for enhancing the local concentration of CO_2_ at the electrode interface.

**Figure 5:**
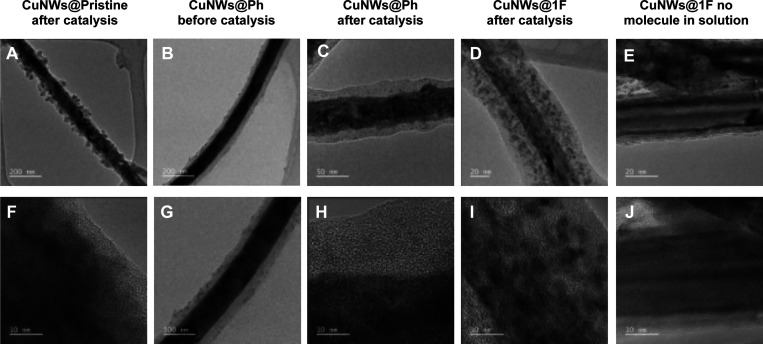
Ex situ TEM and HR-TEM analysis of CuNWs electrodes. A) TEM image of **CuNWs@Pristine** showing surface corrosion after the catalysis test at −1.0 V vs RHE. B) TEM image of **CuNWs@Ph** displaying the presence of an organic overlayer. C) TEM image of **CuNWs@Ph** displaying the presence of an organic overlayer after the catalysis test. D) TEM image of **CuNWs@1F** after electrolysis, indicating a uniform organic coating. E) TEM image of **CuNWs@1F** tested in the absence of **1F** in solution during electrolysis. F) HR-TEM image of a single CuNW from the **CuNWs@Pristine** sample post electrolysis. G) Magnified TEM image of a single CuNW from **CuNWs@Ph** before electrolysis. H) HR-TEM image of a single CuNW from **CuNWs@Ph** post electrolysis. I) HR-TEM image of a single CuNW from **CuNWs@1F** post electrolysis. J) HR-TEM detail of **CuNWs@1F** (no **1F** in solution), showing a thinner and discontinuous organic layer compared to (I).

### 3.2 DFT Calculations

The peculiar catalytic behavior of **CuNWs@OMe**, which exhibited 100% selectivity toward H_2_ production, suggests a role for the Lewis basicity of the pyrazine nitrogens, enhanced by the increased negative charge density injected into the aromatic rings. To gain molecular-level insight into the specific interactions among interfacial water molecules, the functionalized organic shell, and the environmental hydrophobicity, we performed density functional theory (DFT) calculations. These simulations employed single-monomer units as representative models for the local chemical environments of the corresponding oligomeric shell. Multiple possible binding configurations for water molecules on the monomer surfaces were systematically explored, and the four most energetically favorable adsorption sites were identified, as shown in Figure 6A. Among these, sites (1) and (2) were common to all molecular derivatives, while sites (3) and (4), both involving the phenazine moiety, were accessible only to derivatives bearing **OMe**, **Me**, **H**, **Cl**, **2F**, and **1F** substituents. The corresponding water binding energies across different molecules are reported in Figure 6B. Interestingly, substituent variation had a minimal impact on the strength of water–surface interactions. Across all derivatives, the pyrazine ring consistently emerged as the most favorable adsorption site, reinforcing its central role in mediating interfacial water interactions. Notably, the configuration in which a water molecule bridges the two nitrogen atoms of the pyrazine ring, site (4) was found to be less thermodynamically stable, suggesting limited favorability for such bidentate binding. The methoxy-substituted derivative exhibited a markedly different interaction profile, showing significantly altered binding energies compared with the other systems for all four sites. In particular, site (3), adjacent to the phenazine nitrogen atom, displayed a strong affinity for water, with a binding energy of −0.43 eV. This enhanced interaction is associated with the electron-donating nature of the methoxy group that increases the Lewis basicity of the neighboring pyrazine nitrogen atoms, thereby strengthening the hydrogen bonding with water molecules. Importantly, these computational insights provide a compelling explanation for the experimentally observed behavior of the **CuNWs@OMe** catalyst, especially its enhanced activity toward the HER. The stronger water–surface interactions, indeed, likely lead to higher local proton availability and facilitate the hydrogen evolution reaction, ultimately reducing CO_2_RR selectivity.

**Figure 6:**
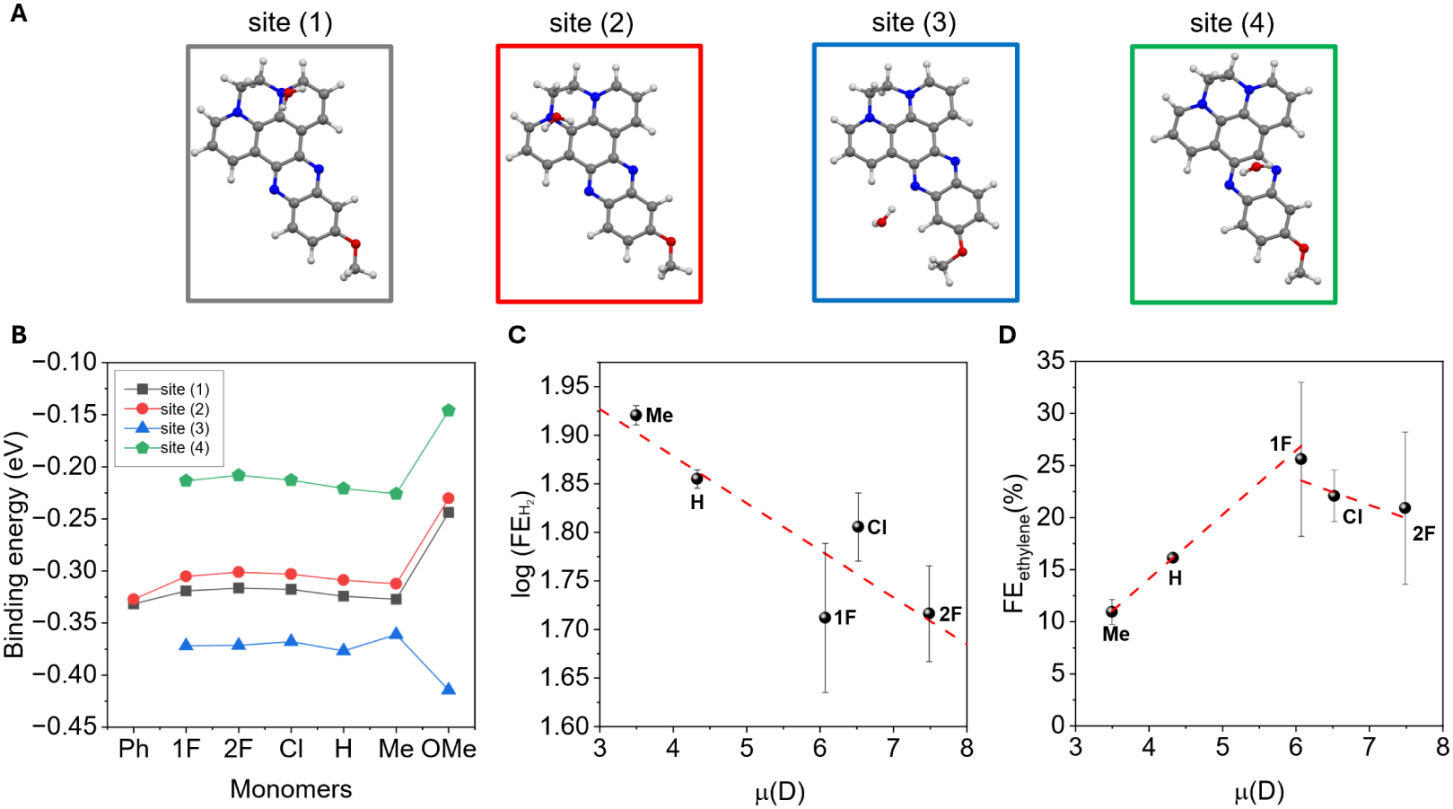
A) Optimized binding configurations of a water molecule adsorbed on representative sites of **Ph**, **F**, **2F**, **Cl**, **H**, **Me**, and **OMe** monomers, as identified by DFT calculations. B) Corresponding water binding energies at the most favorable adsorption sites for each monomer. C) and D) Correlation between the logarithm of the experimentally measured H_2_ FE (C) and ethylene FE (D) and the molecular electric dipole moments computed via DFT.

As a second descriptor to capture the influence of substituents and further elucidate their impact on catalytic selectivity, the molecular electric dipole moments (μ) of the monomer units were calculated. This parameter serves as a proxy for the ability of functional groups to modulate the local electrostatic environment at the catalyst–electrolyte interface.

### 3.3 Correlating Experimental and Calculated Data

A clear correlation was observed between the electric dipole moment of the organic-shell molecular precursor and the logarithm of the H_2_ FE, as illustrated in Figure 6C. Specifically, monomers with higher dipole moments, typically associated with electron-withdrawing substituents, exhibited lower H_2_ evolution and higher selectivity toward CO_2_ reduction. This trend could be ascribed to the ability of more polar molecules to generate stronger local electric fields at the catalyst surface, potentially stabilizing polarizable CO_2_-derived intermediates while simultaneously disfavoring proton reduction pathways. The methoxy-substituted derivative was excluded from this correlation as its pronounced hydrophilic character introduces distinct interfacial interactions with water that deviate from the trend observed for the other monomers. Importantly, this deviation underscores the multifactorial nature of selectivity control, where both electronic and solvation effects must be considered in tandem to fully understand and guide the catalytic behavior. The suppression of H_2_ evolution by our hybrid electrocatalyst leads to enhanced formation of C_2_ products, with ethylene emerging as, by far, the dominant species. A strong local electric field could indeed contribute to orienting adsorbed species, facilitating bonding geometries that are favorable for coupling, for example, parallel alignment of *CO for dimerization, or to affect transition states, lowering activation barriers for multicarbon formation steps. Similarly to a Volcano plot, Figure 6D shows the ethylene FE vs μ trend, revealing that **1F** is the best-performing molecule, thus further confirming the multifactorial and sometimes counterbalancing effects that electronic properties on the interfacial environment exert on this complex reaction. The correlation plots in Figure 6C–D were built exclusively from the homologous series of *dppz* that share the same backbone and vary only in terminal polarity. **CuNWs@Ph** features an aromatic scaffold with distinct polarizability; therefore, including it would mix scaffold effects with the dipole descriptor and blur the trend.

In terms of technological relevance, while the present study provides fundamental insights into the activity and selectivity of **CuNWs@X** catalysts in H-cell configurations, future efforts should focus on their translation to industrially relevant architectures. The limited CO_2_ solubility and associated mass transport constraints in aqueous H-cells restrict achievable current densities, highlighting the need to integrate CuNWs into gas-fed systems, such as GDE-based flow cells. This transition requires addressing challenges related to catalyst stability under high current densities,^65^ effective immobilization of CuNWs on porous substrates, hydrophobicity, and optimization of catalyst layer thickness to ensure balanced water management, gas accessibility, and mechanical integrity. Work is ongoing to adapt the nanowire morphology to porous supports and assess their performance under realistic CO_2_ electrolysis conditions.

## 4 Conclusions

This study introduces a modular hybrid catalyst architecture that integrates a conductive Cu nanowire core with a tunable organic molecular shell, enabling precise modulation of the catalytic microenvironment for CO_2_ electroreduction. By systematically varying the electronic properties of different substituent groups on novel phenazine derivatives, we finely controlled the interfacial hydrophobicity, the hydrogen bonding, and the charge distribution, factors shown to critically govern product selectivity. Faradaic efficiencies for ethylene reached up to 41%, with electron-withdrawing groups enhancing C–C coupling, probably via electric dipole-driven stabilization of *CO intermediates, while electron-donating groups promoted HER through increased proton availability. Our findings, supported by DFT calculations, decouple catalytic performance from the intrinsic properties of the metal core, highlighting the power of interfacial molecular engineering to steer reaction pathways. Notably, our results challenge the notion that spatial confinement alone dictates selectivity, emphasizing instead the central role of local chemical interactions, particularly through hydrophobicity, hydrogen bonding, and electronic effects, without altering the underlying catalytic material. Beyond mechanistic insight, this work lays the foundation for a broadly applicable design strategy in the CO_2_RR, demonstrating that selective and scalable carbon valorization is achievable through rational surface functionalization of the CuNWs–organic platform. By decoupling surface chemistry from bulk catalyst composition, this strategy paves the way for practical, modular CO_2_ conversion technologies with tunable performance profiles tailored to specific value-added products. Importantly, the hybrid CuNWs–organic platform demonstrated here could offer a scalable, low-cost, and solution-processable route to selective CO_2_ electroreduction, addressing key barriers to industrial translation. A straightforward implementation of a flow-through setup based on a self-standing, modified CuNWs-based membrane is anticipated to enable practical, high-performance CO_2_ conversion.

## Supplementary Material



## Notes

Synthetic procedures and additional material characterization details including SEM and STEM images, EDX, XRD, mass and Raman spectra, cyclic voltammetry, EIS, and chronoamperometry data (PDF)
