# Supplementary material for: Modulating C 2 Selectivity in CO 2 Electroreduction through Molecular Surface Engineering of Copper Nanowires
Source: ACS Appl Energy Mater. 2025 Nov 11;8(22):16818–28. doi: 10.1021/acsaem.5c02727 (PMC12648469; doi:10.1021/acsaem.5c02727)
Supplement: Supplementary file 1 [file ae5c02727_si_001.pdf]

Supporting Information

Modulating C_2 Selectivity in CO_2 Electroreduction via Molecular Surface Engineering of Copper Nanowires

Andrea Conte,[a] Chiara Alberoni,[a] Silvia Carlotto,[a] Marco Baron,[a] Sara Bonacchi,[a] Alessandro Aliprandi,*[a] and Sabrina Antonello*[a]

[a] Department of Chemical Sciences, University of Padova, 35131, Via F. Marzolo, Padova, Italy.

Corresponding Author

* Alessandro Aliprandi, Sabrina Antonello.

Contents list

- **Figure S1**. A) SEM image of CuNWs. The inset shows the histogram of sampled NWs. B) General protocol for the fabrication of CuNWs electrode.
  Pag.5
- **Figure S2.** Activation of CuNWs glass electrode using a constant potential for 60s at -1.3 V vs RHE in 1M $KHCO_3$ solution in ambient conditions
  Pag.5
- **Figure S3.** Cyclic voltammetry of CuNWs@Pristine in 1M NaOH.
  Pag.6
- **Figure S4**. A) Cyclic voltammetries of CuNWs in 1 M $KHCO_3$, in the absence (black line) and in the presence of **1F**. B) Chronoamperometry of **1F** at -0.6 V vs RHE recorded during additive deposition procedure.
  Pag.6
- **Figure S5.** SEM image of zones investigated by means of EDX analysis.
  Pag.7
- **Figure S6.** EDX spectrum of spot 24 highlighted in Figure S17. The presence of O, Na, Mg, Si, Ca, was due to the nature of holder that was glass.
  Pag.7
- **Table S1.** Elemental composition of investigated areas relative to Figure S5.
  Pag.8
- **Figure S7.** STEM image of **CuNWs@1F** post electrolysis at -1.0V vs RHE in 1 M $KHCO_3$. B) Copper EDS element map of **CuNWs@1F**. C) Carbon EDS element map of **CuNWs@1F**. D) Nitrogen EDS element map of **CuNWs@1F**. E) Oxygen EDS element map of **CuNWs@1F**.
  Pag.8
- **Figure S8.** STEM image of **CuNWs@1F** (no molecules in solution) post electrolysis at -1.0V vs RHE in 1 M $KHCO_3$. B) Copper EDS element map of **CuNWs@1F**. C) Carbon EDS element map of **CuNWs@1F**. D) Nitrogen EDS element map of **CuNWs@1F**. E) Oxygen EDS element map of **CuNWs@1F**.
  Pag.9

- **Figure S9.** Raman spectra of CuNWs@X.
Pag.9
- **Figure. S10.** ESI mass spectra of monomers and oligomers. The oligomers recovered from reaction solutions.
Pag.10
- **Table S2** Mass of organic additives and their oligomers. N is equal to the number of monomers composing the oligomers.
Pag.11
- **Figure S11.** Different possible oligomers structures.
Pag.11
- **Figure S12.** Blanck cyclic voltammetry of **CuNWs@Ph** in 1M NaOH under Ar saturated solution, before catalysis test.
Pag.12
- **Figure S13**. Constant potential electrolysis (CPE) experiment of **CuNWs@Ph** and **CuNWs@1F** at -1.0 V vs RHE in 1 M $KHCO_3$ for 5400 s.
Pag.12
- **Figure S14.** Partial current density for each chemical species produced in CO_2RR using **CuNWs@X (X** = **Ph-diket**, **OMe**, **Pristine**, **H**, **Cl**, **2F**, **1F**, **Ph)** electrodes at -1.0 V vs RHE in 1 M $KHCO_3$.
Pag.13
- **Figure S15**. Stability test of **CuNWs@Ph**, **CuNWs@2F**, and **CuNWs@OMe.** Only ethylene and hydrogen are shown as main products.
Pag.13
- **Table S3.** Faradaic efficiencies of additional catalytic tests to analyze the effect of electrolyte concentration, absence of CO_2, and metal catalyst morphology.
Pag.14
- **Figure S16.** EIS spectra of **CuNWs@Pristine**, **CuNWs@Ph-diket**, **CuNWs@Ph**, and **CuNWs@OMe** electrodes, recorded at -0.6 V vs RHE, from 100 KHz to 1 Hz. The electrolyte was 1 M $KHCO_3$.
Pag.14
- **Figure S17.** Equivalent circuit proposed for CuNWs electrodes: A) **CuNWs@Pristine** B) **CuNWs@Ph-diket** C) **CuNWs@Ph**, D) **CuNWs@OMe**.
Pag.15
- **Table S4.** Catalysts performance of recent published works on CO_2RR using engineering surface approach.
Pag.17
- **Figure S18.** Partial current density for each chemical species produced in CO_2RR using **CuNWs@Ph** electrode at different potential in 1 M $KHCO_3$.
Pag.18
- **Figure S19.** Thickness of the organic shell on the CuNWs after the catalytic tests at -1.0 V vs RHE.
Pag.18
- **Figure S20.** A) STEM image **CuNWs@1F** post electrolysis at -1.0 V vs RHE without the use of **1F** during the experiment. (high contrast). B) STEM image **CuNWs@1F** after electrolysis at -1.0 V vs RHE without the use of **1F** during the experiment. (low contrast).
Pag.19
- **Figure S21.** A) STEM image **CuNWs@1F** post electrolysis at -1.0 V vs RHE with the use of 1mM of **1F** during the experiment. (low contrast). B) STEM image **CuNWs@1F** after electrolysis at -1.0 V vs RHE with the use of 1mM of **1F** during the experiment. (high

contrast).
Pag.19

- **Figure S22.** A) Faradic efficiency of **CuNWs@1F** with 1mM **1F** molecule in solution and without. B) SEM of **CuNWs@1F** after electrolysis at -1.0 V vs RHE in 1 M $KHCO_3$ C) SEM of **CuNWs@1F** after electrolysis at -1.0 V vs RHE in 1 M $KHCO_3$, no molecule in solution during the electrolysis.
Pag.20
- **Figure S23.** Cyclic voltammetry of **CuNWs@Pristine** and **CuNWs@Cl** in 1 M $KHCO_3$ under Ar conditions.
Pag.21
- **Figure S24.** Cyclic voltammetry **of CuNWs@Pristine** electrode and **CuNWs@OMe** electrode in 1 M $KHCO_3$ under Ar conditions.
Pag.21
- **Figure S25**. Comparison between literature benchmarks and this work for CO_2RR selectivity. The plot displays the faradaic efficiency (FE) of ethylene versus ethanol reported in various literature studies (black dots). The red pentagonal markers represent the performance of **CuNWs@Ph** and **CuNWs@2F**.
Pag.22
- **Figure. S26.** SEM images of CuNWs electrodes after electrolysis test: A) **Ph**, B) **Ph-diket**, C) **2F**, D) **1F**, E) **Cl**, F) **H**, G) **Me**, H) **OMe**.
Pag.23
- **Figure S27.** A) SAED CuNWs@1F, and B) CuNW@1F without molecule in solution during the electrolysis.
Pag.23
- **Figure S28**. XRD of **CuNWs@Pristine** and **CuNWs@1F** post catalysis test at -1.0 V vs RHE.
Pag.24
- **Figure S29.** HR-TEM image of **CuNWs@1F** (no additive in solution during electrolysis) after catalytic test -1.0V vs RHE. The magnification on the right shows the crystal planes features.
Pag.24
- **Figure S30.** Example of NMR spectrum of liquid phase of electrolysis experiment carried out using **CuNWs@Ph** electrode at -1.1 V vs RHE.
Pag.25
- **Scheme S1.** Synthesis of di-pyridyl-phenazil-disubstituted derivatives.
Pag.25
- **Scheme S2.** Synthesis of alkylate phenanthroline and DPPZ derivatives.
Pag.26
- **Figure S31.** ^{1}H NMR (D_2O, 298 K) of **DPPZ2F**.
Pag.28
- **Figure S32.** ^{1}H NMR ($CDCl_3$, 298 K) of **2F.**
Pag.28
- **Figure S33.** ^{1}H NMR (D_2O, 298 K) of **DPPZ1F.**
Pag.29
- **Figure S34.** ^{1}H NMR ($CDCl_3$, 298 K) of **1F**.
Pag.29
- **Figure S35.** ^{1}H NMR (D_2O, 298 K) of **DPPZCl**.
Pag.30
- **Figure S36.** ^{1}H NMR ($CDCl_3$, 298 K) of **Cl.**
Pag.30

- **Figure S37.** ^{1}H NMR (D_2O, 298 K) of **DPPZ.** Pag.31
- **Figure S38.** ^{1}H NMR ($CDCl_3$, 298 K) of **H**. Pag.31
- **Figure S39.** ^{1}H NMR (D_2O, 298 K) of **DPPZCH$_3$.** Pag.32
- **Figure S40.** ^{1}H NMR ($CDCl_3$, 298 K) of **Me**. Pag.32
- **Figure S41.** ^{1}H NMR (D_2O, 298 K) of **DPPZOCH$_3$.** Pag.33
- **Figure S42.** ^{1}H NMR ($CDCl_3$, 298 K) of **OMe.** Pag.33
- **Figure S43.** ^{1}H NMR (D_2O, 298 K) of **Ph.** Pag.34

References Pag.34

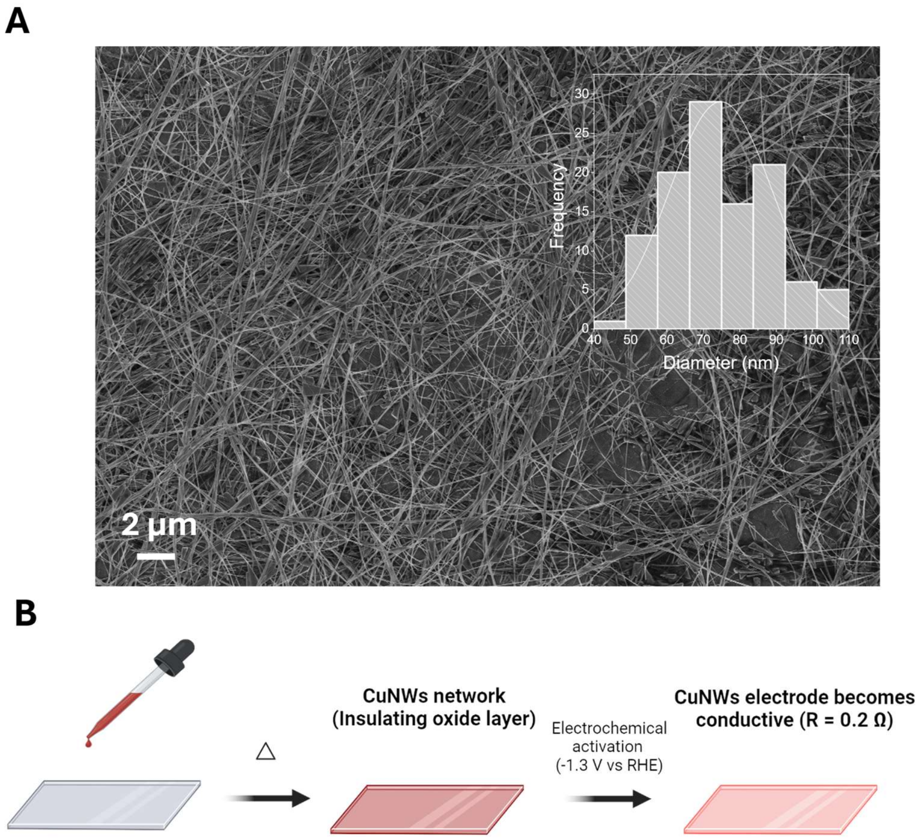

Figure S1. A) SEM image of CuNWs. The inset shows the histogram of sampled NWs. B) General protocol for the fabrication of CuNWs electrode.

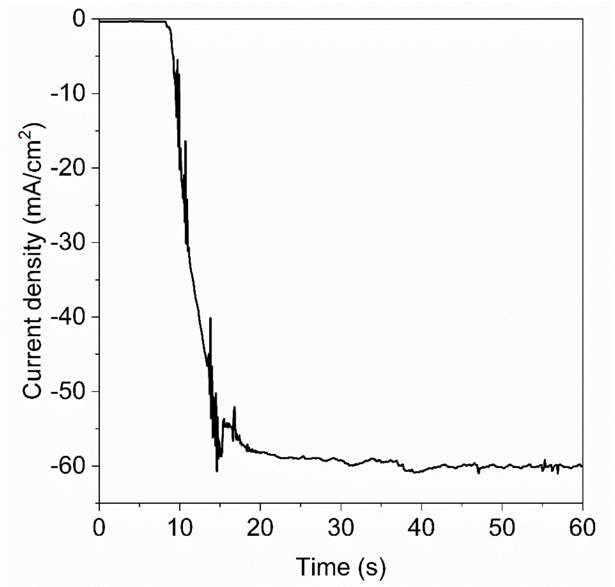

Figure S2. Activation of CuNWs glass electrode using a constant potential for 60s at -1.3 V vs RHE in 1 M $KHCO_3$ electrolyte solution at ambient conditions.

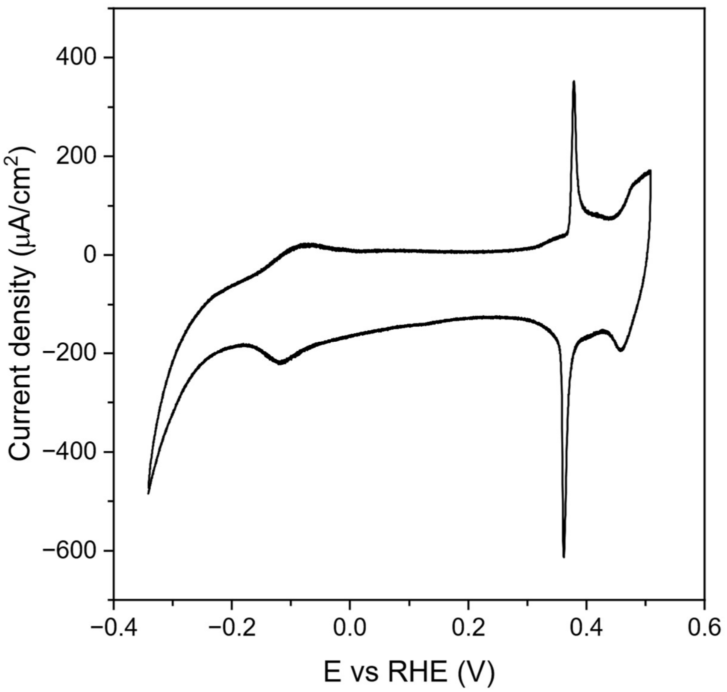

Figure S3. Blank cyclic voltammetry of **CuNWs@Pristine** in 1 M NaOH under Ar saturated solution.

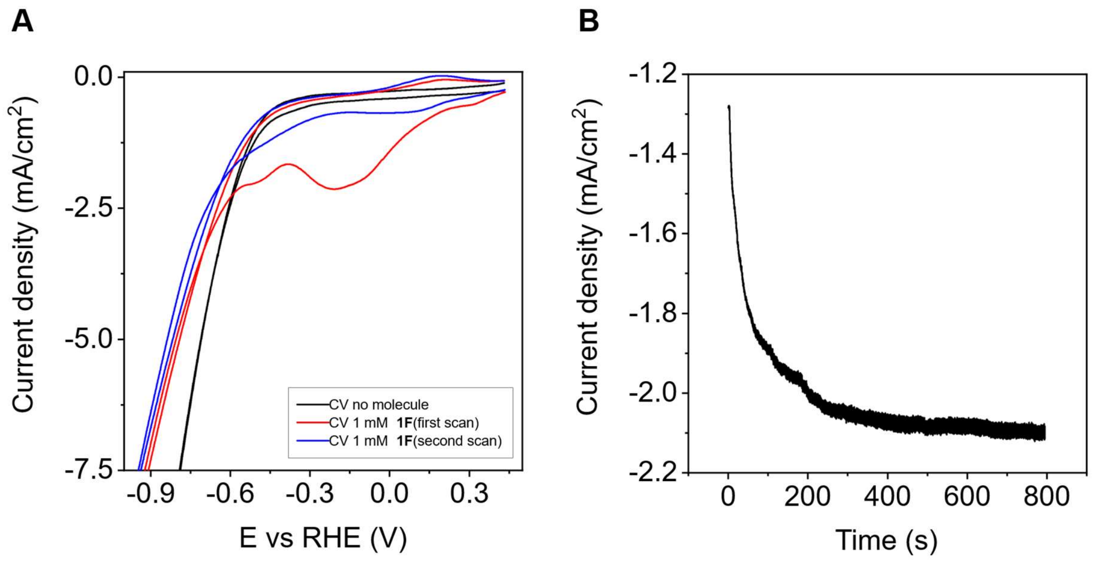

Figure S4. A) Cyclic voltammetries of CuNWs in 1 M $KHCO_3$, in the absence (black line) and in the presence of **1F**. B) Chronoamperometry of **1F** at -0.6 V vs RHE recorded during additive deposition procedure.

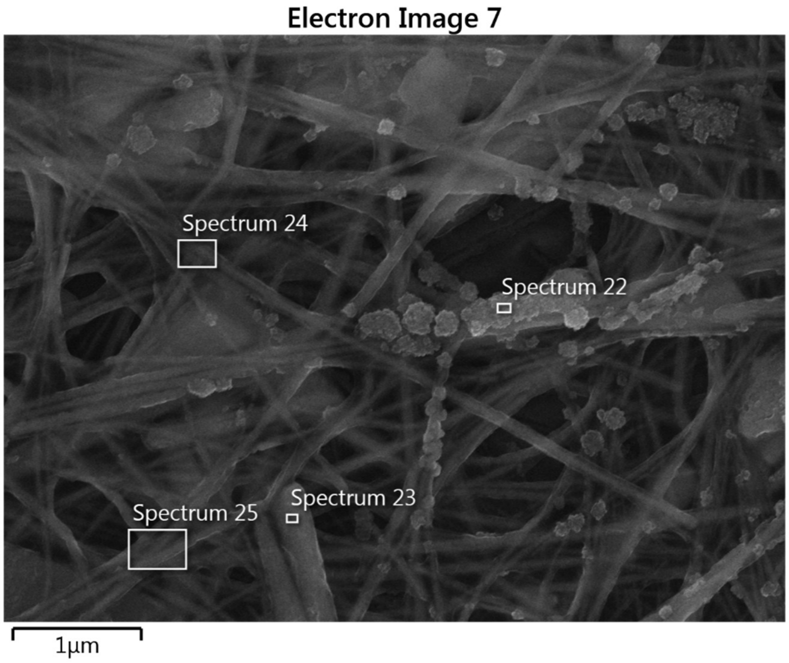

Figure S5. SEM image of CuNWs with highlighted the regions investigated by means of EDX analysis.

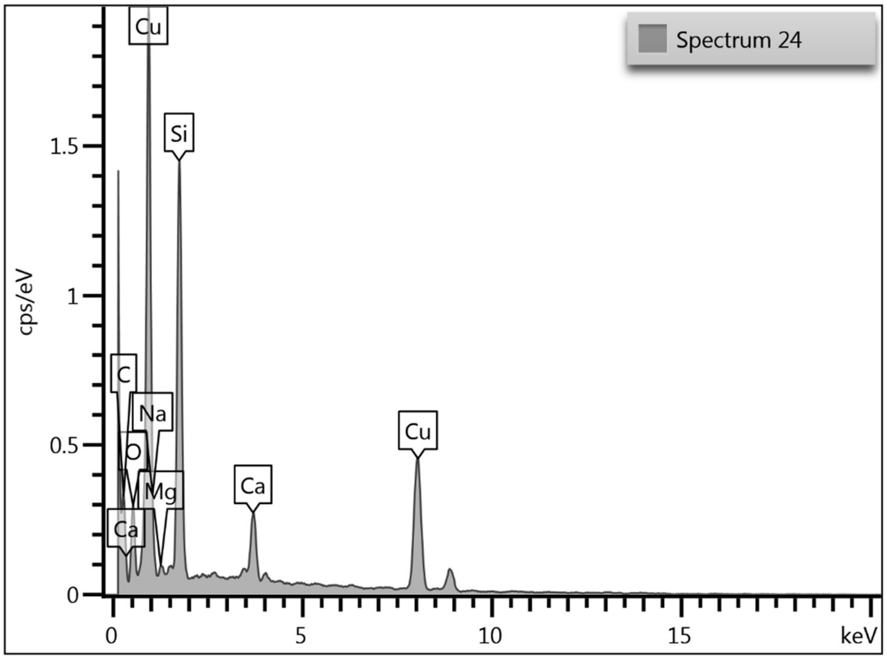

Figure S6. EDX spectrum of spot 24 highlighted in Figure S5. The presence of O, Na, Mg, Si, Ca, was due to the nature of holder that was glass.

Table S1. Elemental composition of investigated areas relative to Figure S5.

Spectrum Label	Spectrum 22	Spectrum 23	Spectrum 24	Spectrum 25
C	17.47	26.35	25.25	21.38
O	16.48	13.71	14.92	19.04
Na	3.52	2.69	2.74	2.97
Mg	1.03	0.97	0.91	1.09
Si	14.56	14.39	16.91	22.35
Ca	2.75	2.87	3.74	5.00
Cu	44.18	39.02	35.52	28.17
Total	100.00	100.00	100.00	100.00

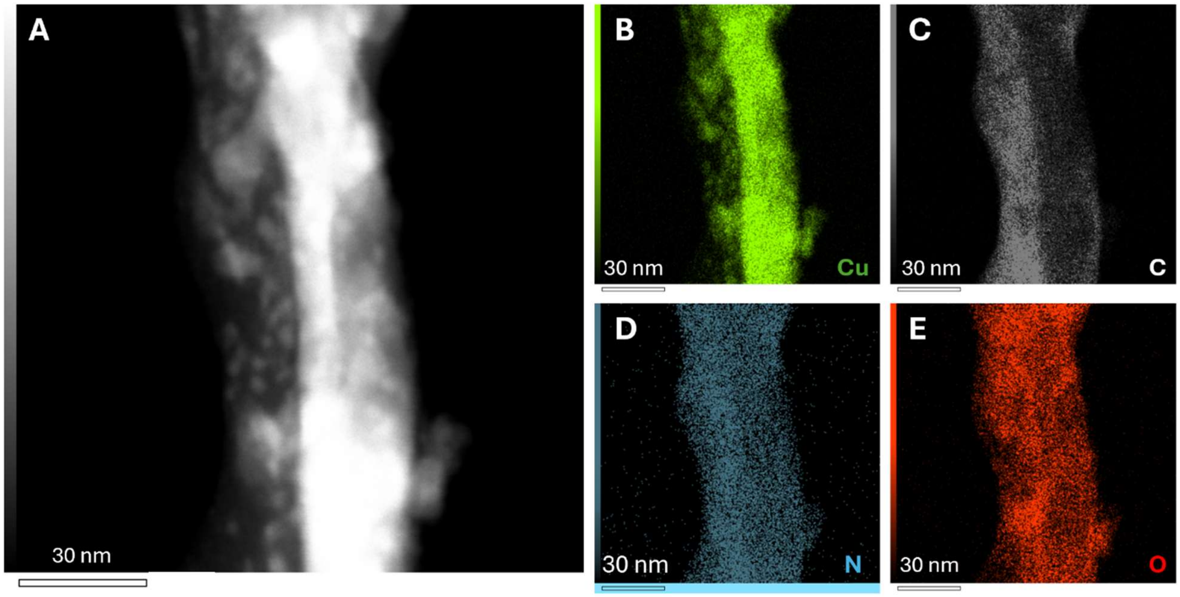

Figure S7. STEM image of **CuNWs@1F** post electrolysis at -1.0V vs RHE in 1 M $KHCO_3$. B) Copper EDS element map of **CuNWs@1F**. C) Carbon EDS element map of **CuNWs@1F**. D) Nitrogen EDS element map of **CuNWs@1F**. E) Oxygen EDS element map of **CuNWs@1F**.

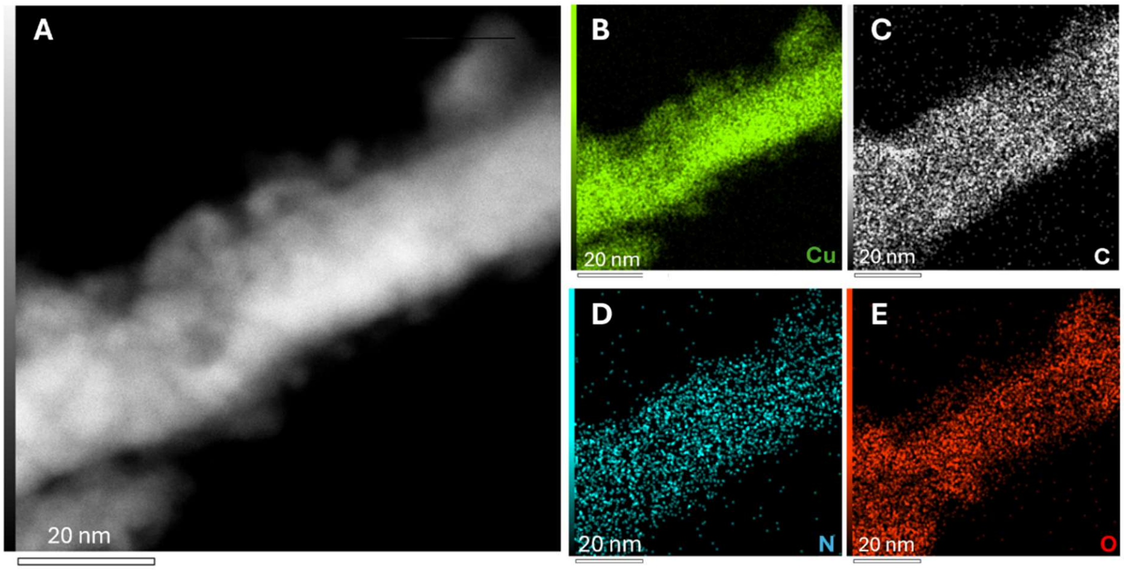

Figure S8. STEM image of **CuNWs@1F** (no molecules in solution) post electrolysis at -1.0 V vs RHE in 1 M $KHCO_3$. B) Copper EDS element map of **CuNWs@1F**. C) Carbon EDS element map of **CuNWs@1F**. D) Nitrogen EDS element map of **CuNWs@1F**. E) Oxygen EDS element map of **CuNWs@1F**.

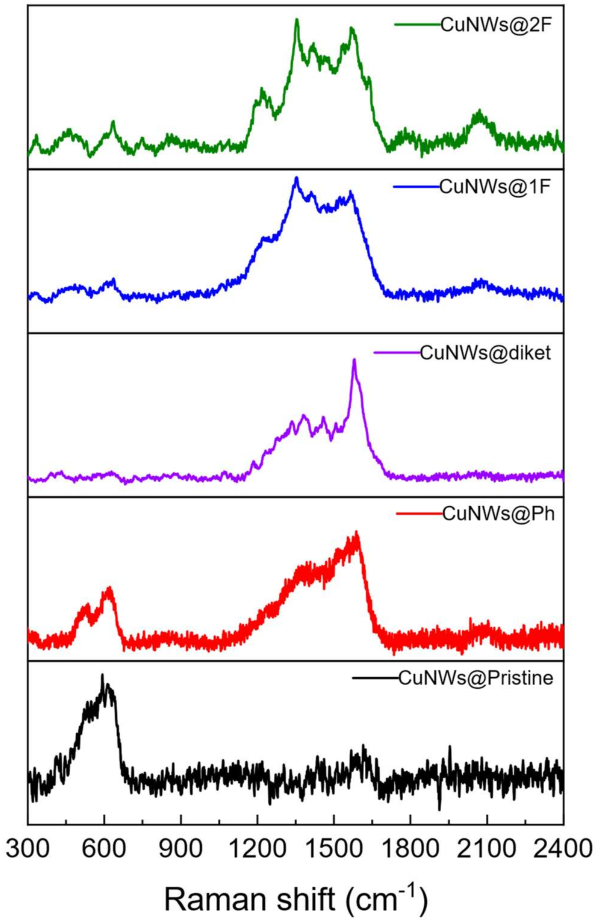

Figure S9. Raman spectra of **CuNWs@X.**

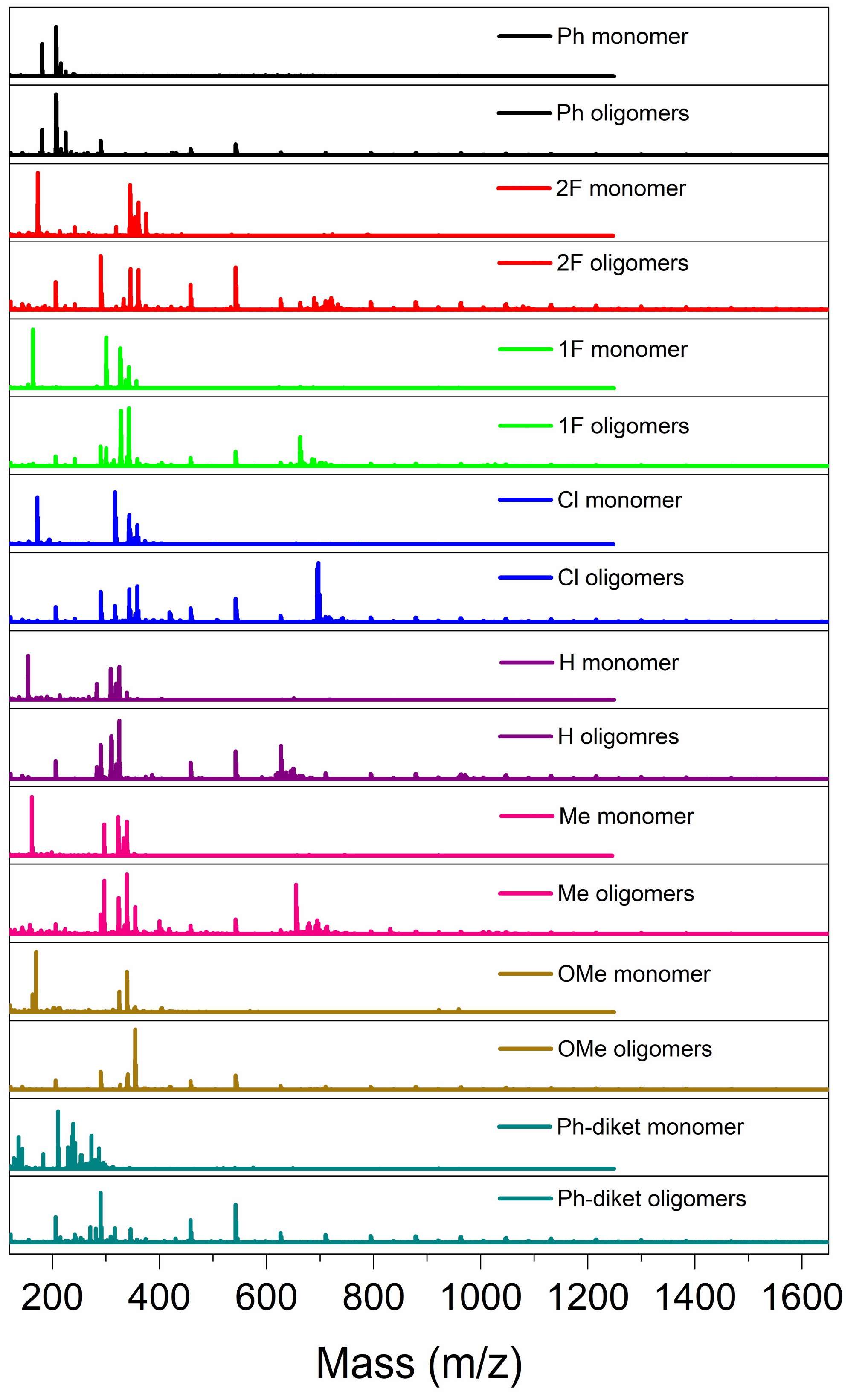

Figure. S10. ESI mass spectra of electroreduced organic additives recovered from reaction solutions.

Table S2. Mass of organic additives and their oligomers. NX refers to the number of monomers that eventually compose the oligomers.

Compound	MW (g/mol)	N1	N2	N3	N4	N5	N6	N7	N8
Ph	368.06	208.26	418.52	837.04	839.04	1049.3	1259.56	1469.82	1680.08
Ph-diket	398.04	238.24	478.48	956.96	958.96	1199.2	1439.44	1679.68	1919.92
1F	488.15	328.35	658.7	1317.4	1319.4	1649.75	1980.1	2310.45	2640.8
2F	506.14	346.34	658.5	1317	1319	1649.25	1979.5	2309.75	2640
Cl	500.18	340.38	682.76	1365.52	1367.52	1709.9	2052.28	2394.66	2737.04
H	470.16	310.36	622.72	1245.44	1247.44	1559.8	1872.16	2184.52	2496.88
Me	484.2	324.4	828.4	1656.8	1658.8	2074	2489.2	2904.4	3319.6
OMe	504.6	344.8	691.6	1383.2	1385.2	1732	2078.8	2425.6	2772.4

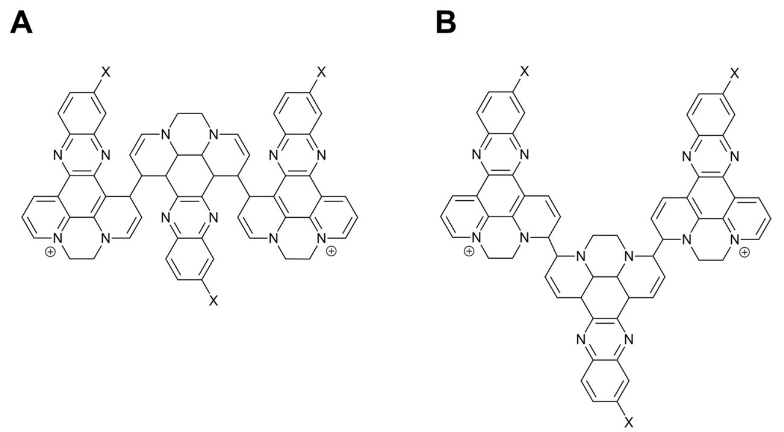

Figure S11. Different possible oligomers structures.

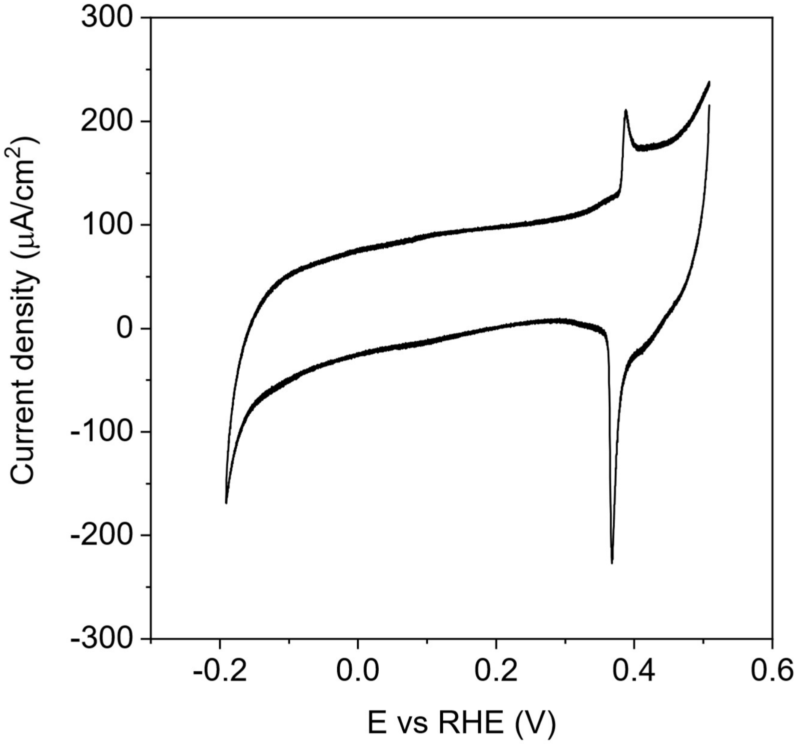

Figure S12. Blank cyclic voltammetry of **CuNWs@Ph** in 1 M NaOH under Ar saturated solution, before catalysis test.

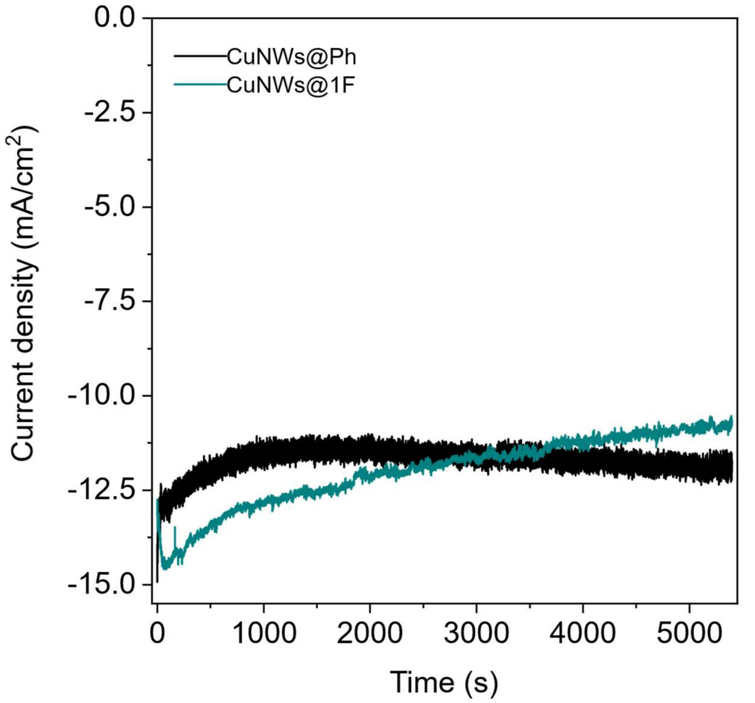

Figure S13. Constant potential chronoamperometry experiments of **CuNWs@Ph** and **CuNWs@1F** at -1.0 V vs RHE in 1 M $KHCO_3$ for 5400 s.

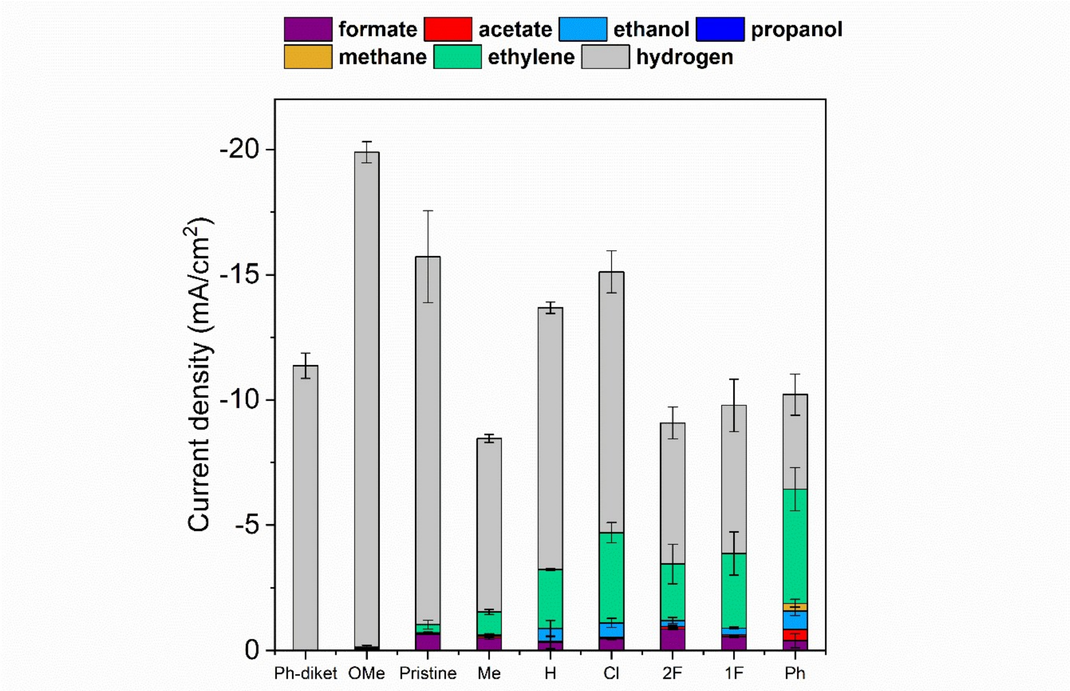

Figure S14. Partial current density for each chemical species produced in CO_2RR using **CuNWs@X (X = Ph-diket**, **OMe**, **Pristine**, **H**, **Cl**, **2F**, **1F**, **Ph)** electrodes at -1.0 V vs RHE in 1 M $KHCO_3$.

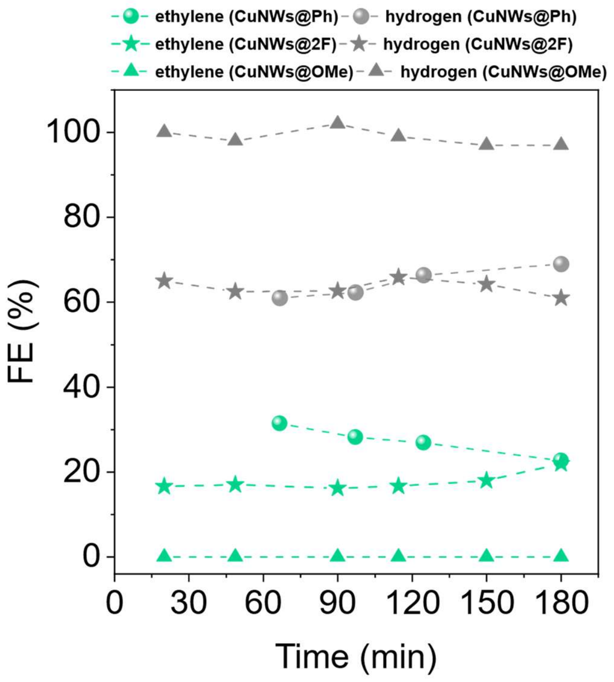

Figure S15. Stability test of **CuNWs@Ph**, **CuNWs@2F**, and **CuNWs@OMe.** Only ethylene and hydrogen are shown as main products.

Table S3. Faradaic efficiencies of additional catalytic tests to analyze the effect of electrolyte concentration, absence of CO_2, and metal catalyst morphology.

Electrode	Conditions	Formate (%)	Acetate (%)	Methane (%)	Ethylene (%)	Ethanol (%)	Propanol (%)	Hydrogen (%)
CuNWs@1F	1 M $KHCO_3$, 1mM 1F, no CO_2	0.1	0.1	1.2	0.3	0.3	0	102.8
CuNWs@2F	0.1 M $KHCO_3$, 1mM 2F	15.9	2.2	0	18.5	8.4	9.5	47.2
Cu bulk (electropolished H_3PO_4, at +3V)	1 M $KHCO_3$	0	0	0.5	0	0	0	100
Cu bulk@Ph	1 M $KHCO_3$, 1 mM Ph	11.2	1.1	8.9	30.6	8.8	5.2	38.2

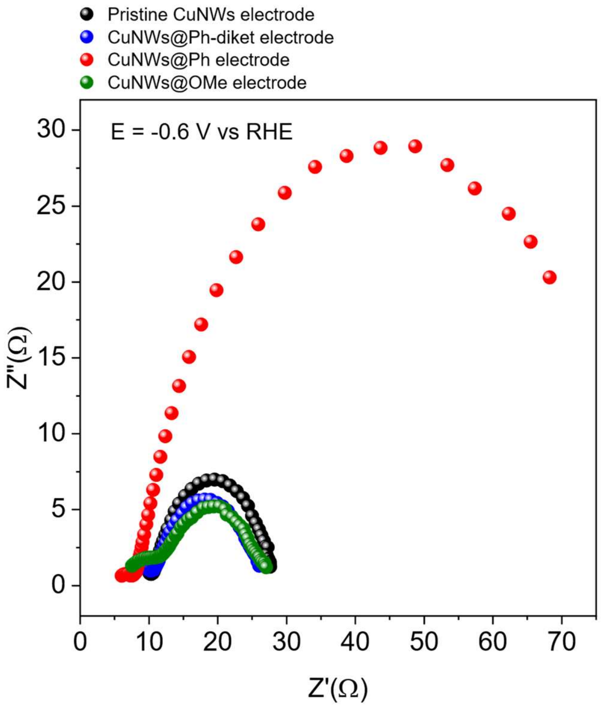

Figure S16. EIS spectra of **CuNWs@Pristine**, **CuNWs@Ph-diket**, **CuNWs@Ph**, and **CuNWs@OMe** electrodes, recorded at -0.6 V vs RHE, from 100 KHz to 1 Hz. The electrolyte was 1 M $KHCO_3$.

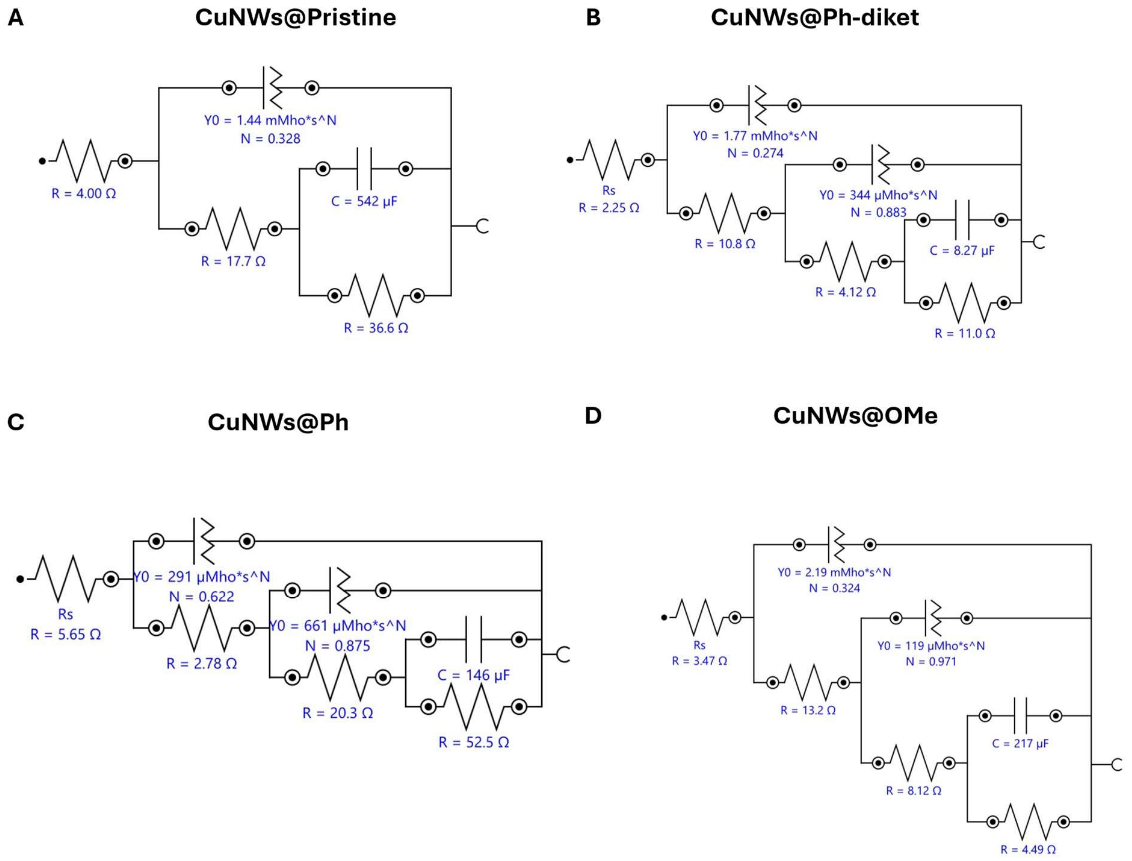

Figure S17. Equivalent circuit proposed for CuNWs electrodes: A) **CuNWs@Pristine** B) **CuNWs@Ph-diket** C) **CuNWs@Ph**, D) **CuNWs@OMe**.

Discussion of EIS results.

The Nyquist plots at −0.6 V vs RHE, read together with the fitted equivalent circuits, can be mapped directly onto the real layered interface of solution → organic shell → copper oxide → Cu nanowires. The hydrophilic Ph-diket coating and the hydrophobic Ph coating produce distinct electrochemical "fingerprints" because they change wetting, ion transport, and charge-transfer pathways at these boundaries. In the spectra, CuNWs@Ph-diket (blue) shows the smallest semicircle, comparable to or slightly smaller than pristine CuNWs (black), while CuNWs@Ph (red) exhibits a much larger semicircle with a pronounced low-frequency tail. Qualitatively, the total polarization resistance follows Ph (red) » Pristine (black) ≈ Ph-diket (blue). This ordering anticipates easier interfacial transport and charge transfer for the hydrophilic film and significant kinetic/transport penalties for the hydrophobic film. Each circuit element has a clear physical meaning. The series resistor Rs is the ohmic drop across the electrolyte and the porous nanowire felt. Each CPE‖R branch represents an interfacial time constant: the outer branch captures the solution–organic interface (wetting, roughness, partial gas coverage), and the inner branch captures the organic–oxide/Cu contact (true charge transfer through or around the film and native oxide). The low-frequency R‖C block lumps finite-length diffusion and porous-film storage—slow transport of ions/CO_2 and gas accumulation in the nanowire network. For pristine CuNWs, Rs is ~4 Ω. A single, strongly non-ideal

CPE (N ≈ 0.33) in parallel with R ≈ 17.7 Ω reflects heterogeneous double-layer formation on a rough Cu/Cu_2O surface with incomplete wetting. A large low-frequency R‖C (R ≈ 36.6 Ω, C ≈ 542 µF) indicates substantial porous transport and gas/liquid storage effects. In Nyquist, this appears as one main arc with a noticeable shoulder at low frequency. For CuNWs@Ph-diket (hydrophilic), Rs drops to ~2.25 Ω, consistent with better ionic percolation through a well-wetted nanowire mat. The outer interface (solution ↔ hydrophilic film) still shows some non-ideality (CPE N ≈ 0.274, R ≈ 10.8 Ω) due to roughness/porosity, but the inner interface (film ↔ oxide/Cu) is nearly ideal (N ≈ 0.883) with a low charge-transfer resistance (R ≈ 4.12 Ω). The low-frequency block is small (R ≈ 11 Ω, C ≈ 8.27 µF), implying weaker storage/diffusion limitations. Consequently, the Nyquist arc is smaller and the low-frequency tail is modest—the hydrophilic film improves wetting and facilitates charge transfer across the film/oxide contact. For CuNWs@Ph (hydrophobic), Rs increases to ~5.65 Ω, indicating poorer wetting and more gas-filled pathways. The outer interface shows non-ideal behavior (N ≈ 0.622) consistent with patchy wetting and bubble coverage, while the inner interface exhibits a much higher charge-transfer resistance (R ≈ 20.3 Ω) despite a reasonably ideal exponent (N ≈ 0.875), showing that the hydrophobic layer acts as a kinetic barrier to water/proton delivery and electron-transfer steps at the oxide/Cu. The low-frequency R‖C is large (R ≈ 52.5 Ω, C ≈ 146 µF), revealing strong finite-length diffusion/accumulation beneath a hydrophobic "skin." In Nyquist terms, this yields the largest semicircle and a pronounced low-frequency tail. Taken together, the hydrophilic Ph-diket coating produces a more homogeneous, better-wetted interface with lower ohmic loss, lower charge-transfer resistance at the film/oxide/Cu contact, and weaker transport limitations. The pristine electrode is dominated by rough native oxide and porous-network effects. The hydrophobic Ph coating imposes both kinetic and transport penalties—higher Rs, higher inner-interface Rct, and stronger low-frequency diffusion/storage—consistent with reduced interfacial water activity and partial gas trapping at the outer surface.

In summary, the fitted circuits rationalize the Nyquist trends: the hydrophilic shell enhances ionic access and charge transfer, the pristine surface sits in between, and the hydrophobic shell introduces significant barriers to both charge and mass transport. This electrochemical picture matches the expected wetting and solvation physics at the solution/organic/oxide/CuNWs junction.

Table S4. Catalysts performance of recent published works on CO_2RR using engineering surface approach.

Catalyst	Conditions	Potential/Current density (V vs RHE)/(mA/cm^2)	FE ethylene (%)	FE ethanol (%)	$FE_{ethylene}$ / $FE_{ethanol}$	Reference
**CuNWs**	0.1 M $KHCO_3$	-1.1	38	15	2.5	[1]Smart mat. 2022,3, 142-150
**Cu mesh**	0.1 M $KHCO_3$	-300 mA/cm^2	40	25	1.6	[2]Nat. Comm. 2022, 13, 3158.
**Cu bulk**	0.1 M $KHCO_3$	-1.2	36	40	0.9	[3]Nat. Comm. 2023, 14, 3575.
**Cu foil**	0.1 M $KHCO_3$	-1.0	2	20	0.1	[4]Angew. Chem. Int. Ed. 2021, 60, 4879-4885.
**Cu foil**	0.1 M $KHCO_3$	-1.0	25	6	4.2	[5]ChemCatChem. 2022, 14, e202200540
**Cu foil (Ph)**	0.1 M $KHCO_3$	-1.07	45	14.6	3.0	[6]Angew. Chemi. Int. ed. 2019, 58, 16952-16958
**Cu foil (tolyl-pyr)**	0.1 M $KHCO_3$	-1.07	40	30	1.3	[6]Angew. Chemi. Int. ed. 2019, 58, 16952-16958
**Cu film**	KCl	-200 mA/cm^2	45	30	1.5	[7]Nat. Comm. 2023, 14, 2387
**CuO nanoparticle**	2 M KOH	-0.56	10	45	0.2	[8]Nat. Comm. 2023, 14, 698
**Cu foil (Ph)**	0.1 M $KHCO_3$	-1.1	64	9	7.1	[9]ACS Appl. Mater. Interfaces 2024, 16, 6562-6568
**CuNWs (Vitamin-C)**	1M KOH	-1.0	55	13	4.2	[10]Nat. Comm. 2024, 15, 192.
**Cu bulk (tolyl-pyr)**	0.1 $KHCO_3$	-1.41	≈ 15	≈ 18	0.83	[11]Angew. Chem. Int. Ed. 2023, 62, e202216102
**CuM_x thin film**	0.1 $KHCO_3$	-1.0	≈ 10	≈ 8	1.25	[12]ACS Cent. Sci. 2021, 7, 1756−1762
**Cu foil polycrystalline (pyr)**	0.1 $KHCO_3$	-1.1	40.8	26.7	1.52	[13]ACS Cent. Sci. 2017, 3, 853−859
**CuNWs@1F**	**1 M $KHCO_3$**	**-1.0**	**26**	**2.7**	**9.6**	**This work**
**CuNWs@2F**	**1 M $KHCO_3$**	**-1.0**	**20.9**	**2.1**	**9.9**	**This work**
**CuNWs@Ph**	**1 M $KHCO_3$**	**-1.0**	**41**	**6.6**	**8**	**This work**
**CuNWs@Cl**	**1 M $KHCO_3$**	**-1.0**	**22.1**	**3.6**	**6.2**	**This work**

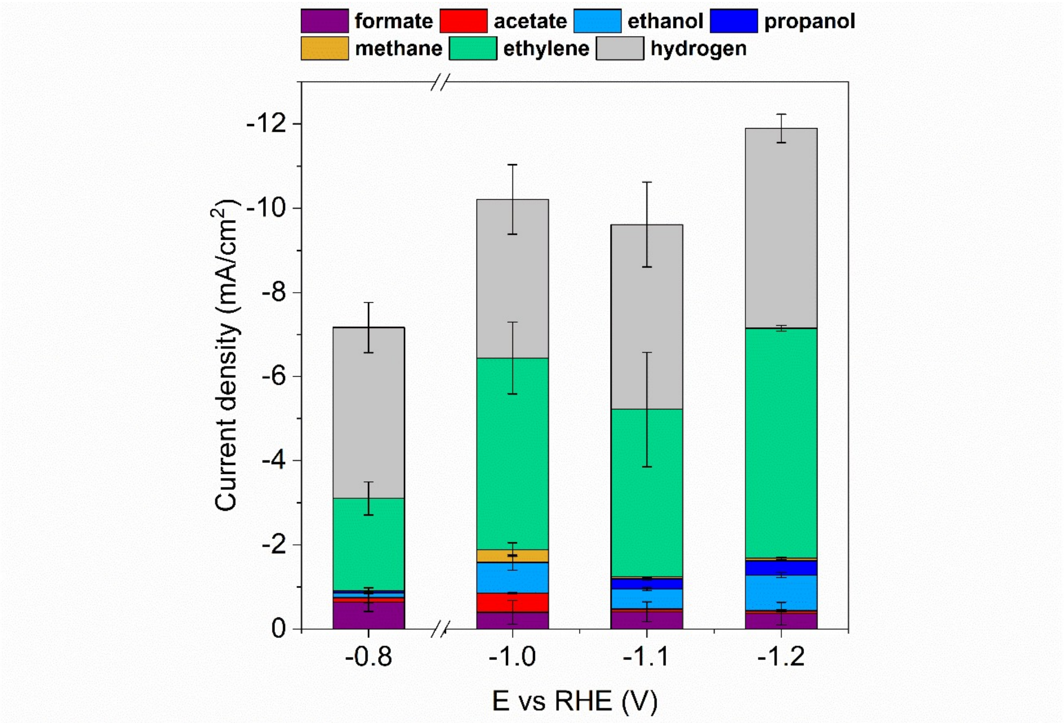

Figure S18. Partial current density for each chemical species produced in CO_2RR using **CuNWs@Ph** electrode at different potential in 1 M $KHCO_3$.

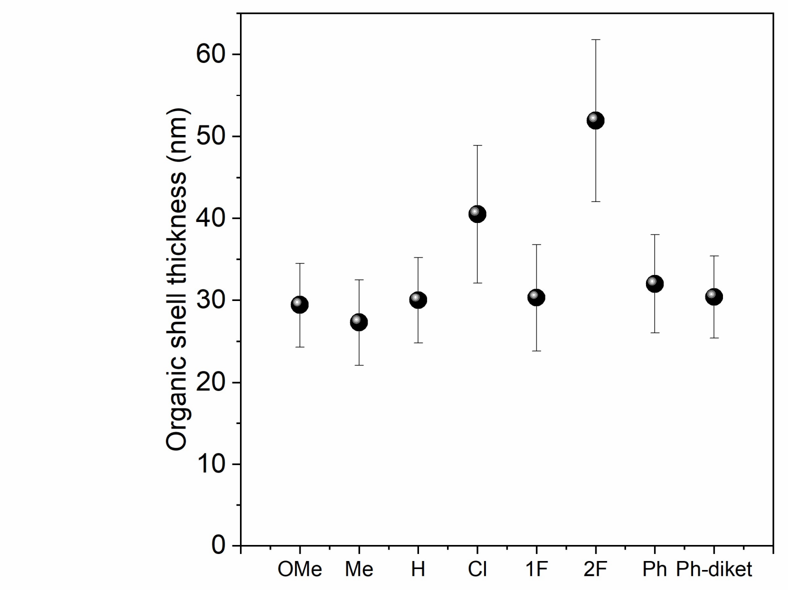

Figure S19. Thickness of the organic shell on the CuNWs after the catalytic tests at -1.0 V vs RHE.

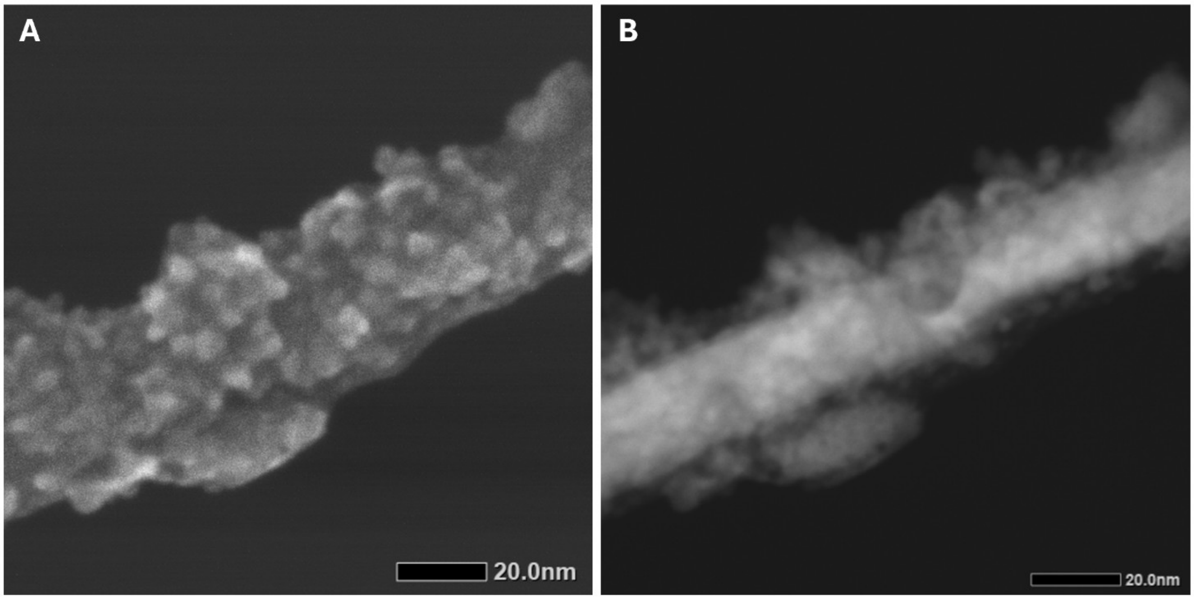

Figure S20. A) STEM image **CuNWs@1F** post electrolysis at -1.0 V vs RHE without the use of **1F** during the experiment. (high contrast). B) STEM image **CuNWs@1F** after electrolysis at -1.0 V vs RHE without the use of **1F** during the experiment. (low contrast).

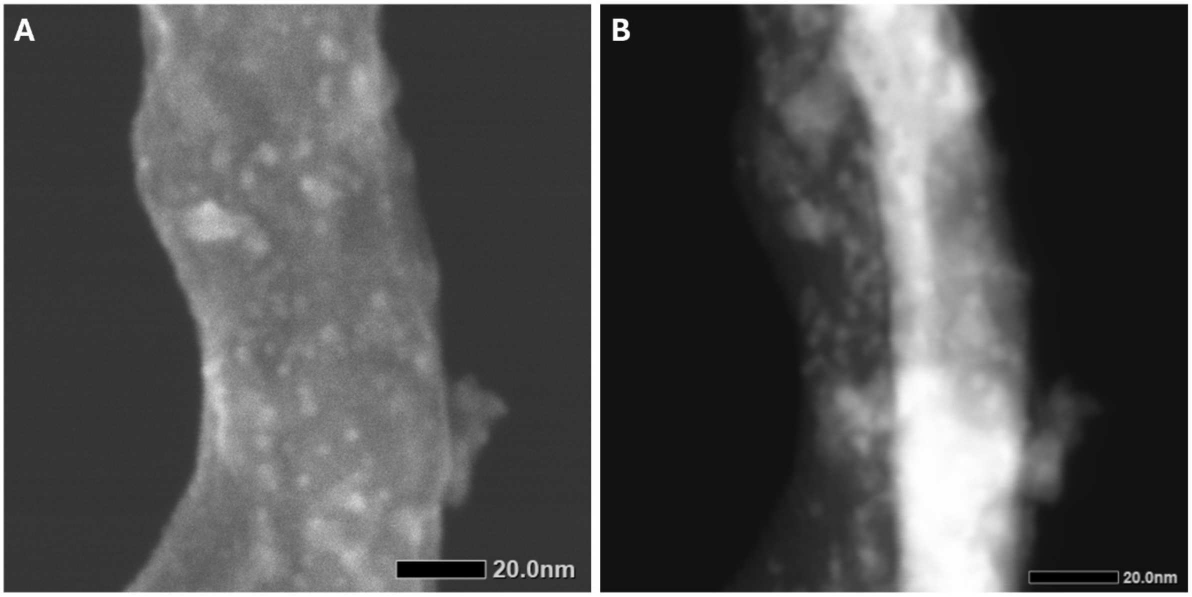

Figure S21. A) STEM image **CuNWs@1F** post electrolysis at -1.0 V vs RHE with the use of 1mM of **1F** during the experiment. (low contrast). B) STEM image **CuNWs@1F** after electrolysis at -1.0 V vs RHE with the use of 1mM of **1F** during the experiment. (high contrast).

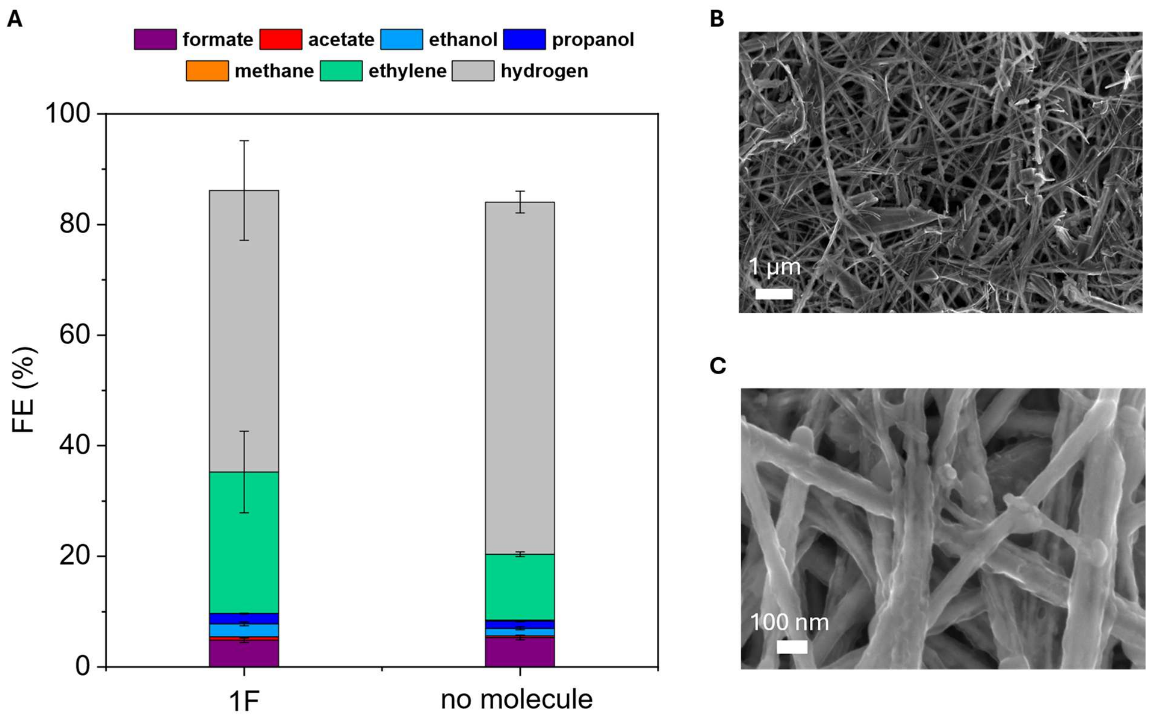

Figure S22. A) Faradic efficiency of **CuNWs@1F** with 1mM **1F** molecule in solution and without. B) SEM of **CuNWs@1F** after electrolysis at -1.0 V vs RHE in 1 M $KHCO_3$ C) SEM of **CuNWs@1F** after electrolysis at -1.0 V vs RHE in 1 M $KHCO_3$, no molecule in solution during the electrolysis.

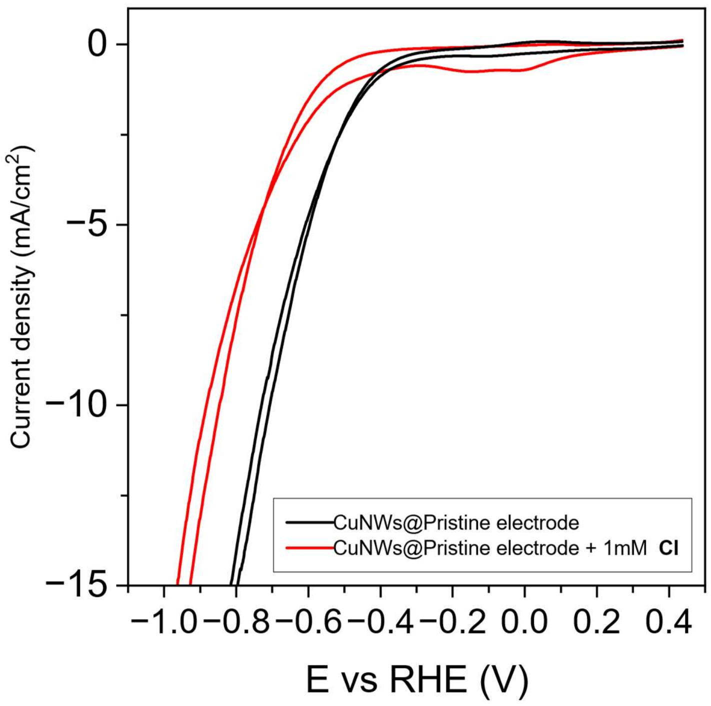

Figure S23. Cyclic voltammetry of **CuNWs@Pristine** electrode and **CuNWs@Cl** electrode in 1 M $KHCO_3$ under Ar conditions.

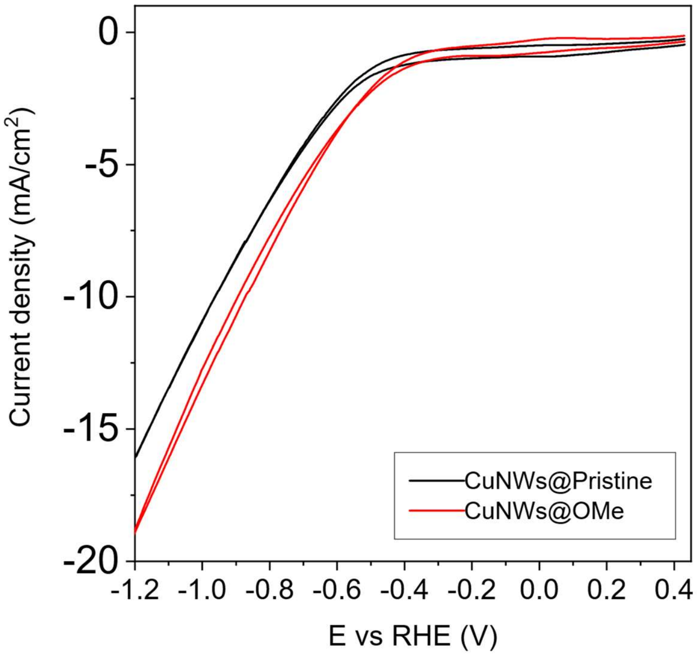

Figure S24. Cyclic voltammetry of **CuNWs@Pristine** electrode and **CuNWs@OMe** electrode in 1 M $KHCO_3$ under Ar conditions.

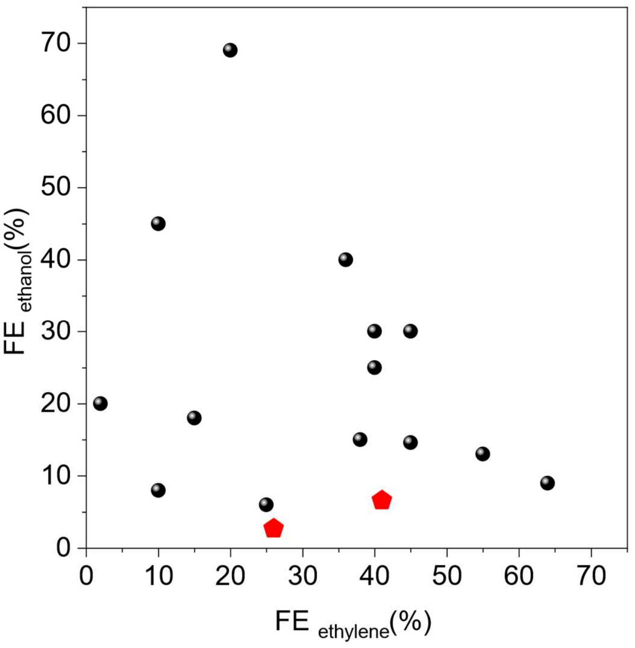

Figure S25. Comparison between literature benchmarks and this work for CO_2RR selectivity. The plot displays the faradaic efficiency (FE) of ethylene versus ethanol reported in various literature studies (black dots). The red pentagonal markers represent the performance of **CuNWs@Ph** and **CuNWs@2F.**

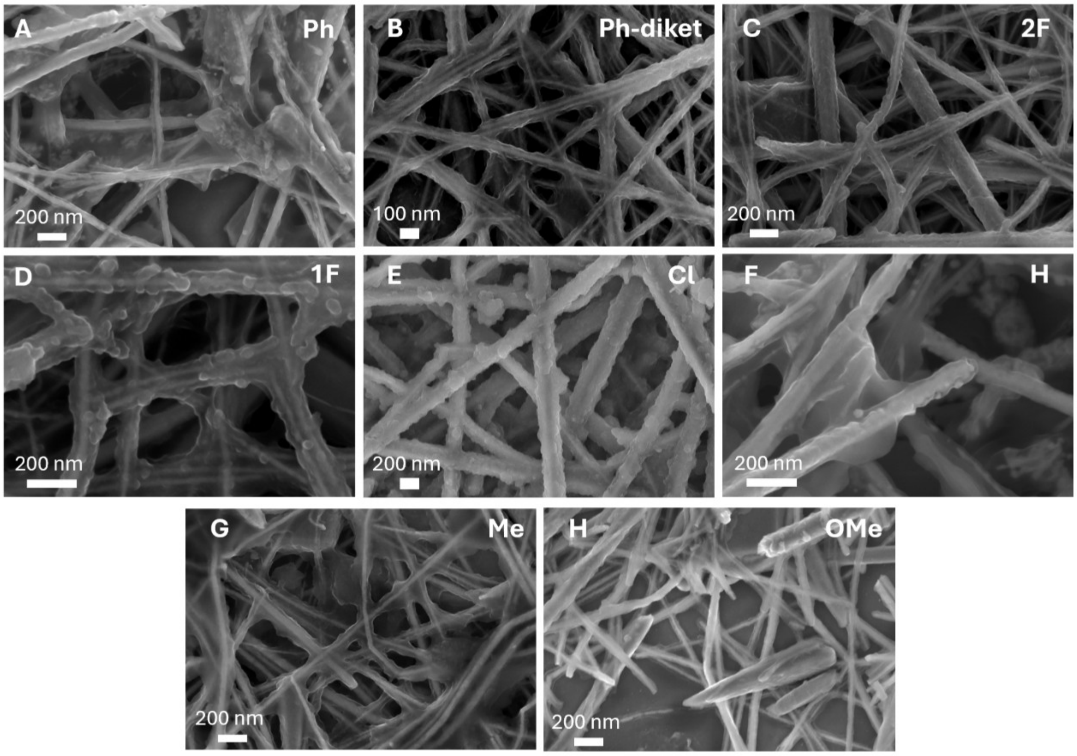

Figure. S26. SEM images of CuNWs electrodes after electrolysis test: A) **Ph**, B) **Ph-diket**, C) **2F**, D) **1F**, E) **Cl**, F) **H**, G) **Me**, H) **OMe**.

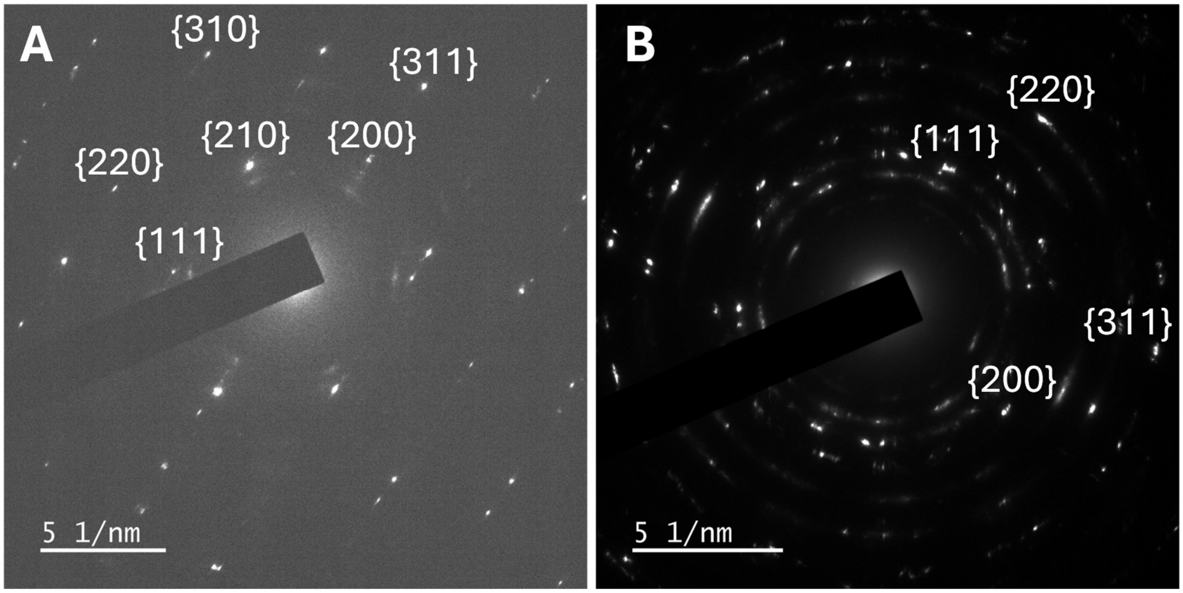

Figure S27. A) SAED **CuNWs@1F**, and B) **CuNW@1F** without molecule in solution during the electrolysis.

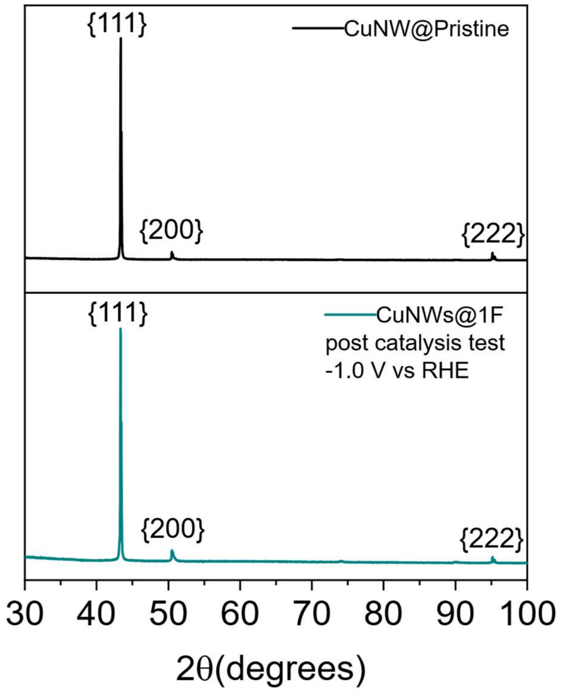

Figure S28. XRD of **CuNWs@Pristine** and **CuNWs@1F** post catalysis test at -1.0 V vs RHE.

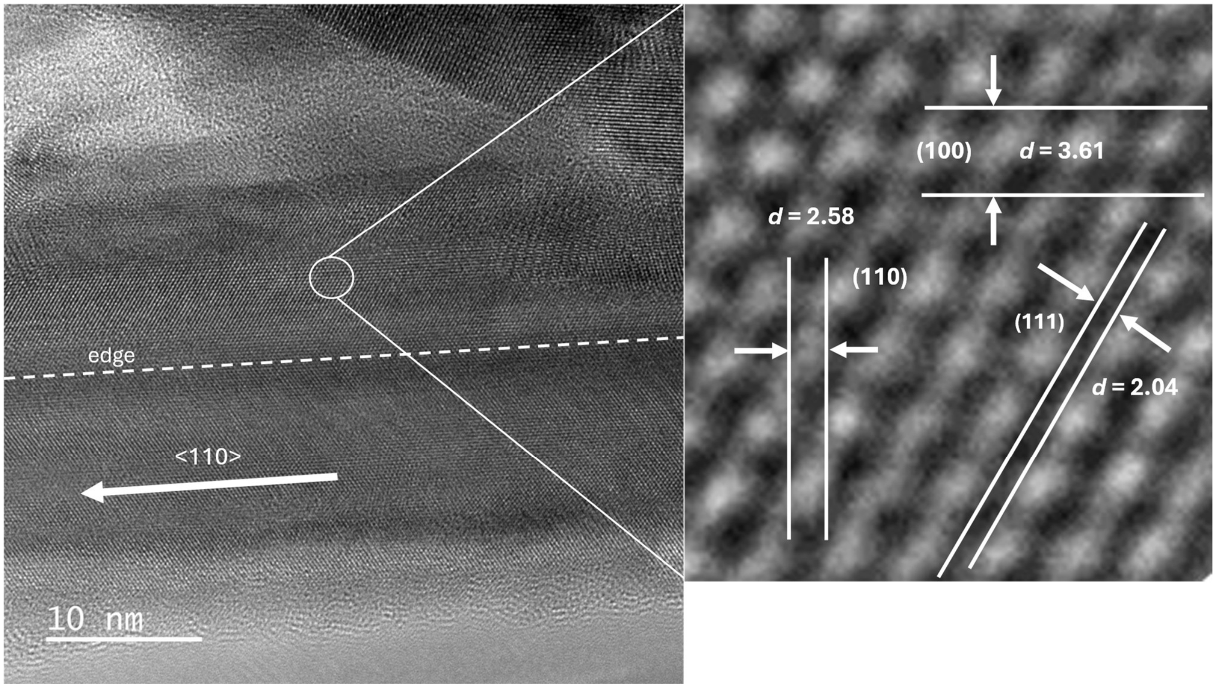

Figure S29. HR-TEM image of **CuNWs@1F** (no additive in solution during electrolysis) after catalytic test -1.0V vs RHE. The magnification on the right shows the crystal planes features.

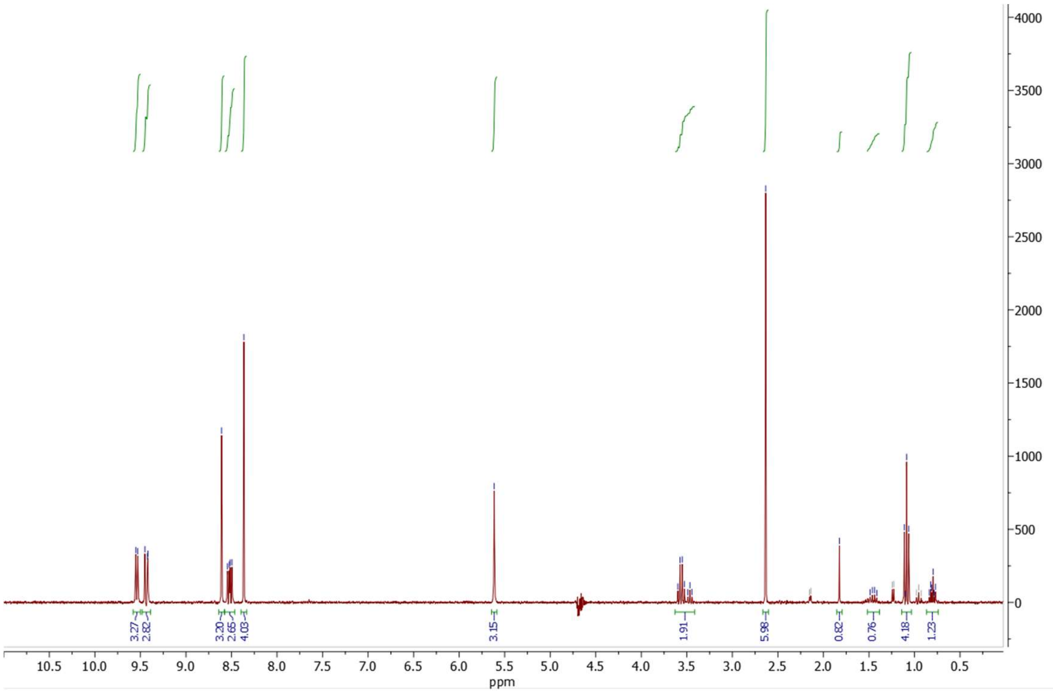

Figure S30. Example of NMR spectrum of liquid phase of electrolysis experiment carried out using **CuNWs@Ph** electrode at -1.1 V vs RHE.

Synthesis routes and procedures.

*Synthesis of Di-Pyridyl-Phenazil-Disubstituted derivatives (**DPPZ**)*

In a flame dried and argon-purged round-bottomed flask equipped with a magnetic stir bar 1,10-phenantroline-5,6-dione (150 mg, 0.71 mmol) were put in acetic acid (15 mL). Then the desired diamine (1.1 eq.) was added, and the suspension refluxing was stirred overnight (*Scheme S1*). Afterward, the mixture was cooled at room temperature and the product was extracted with water (100 mL) and dichloromethane (twice with 100 mL). The organic phase was dried with Na_2SO_4, filtered and dried under vacuum. The powder products were obtained in a high yield.

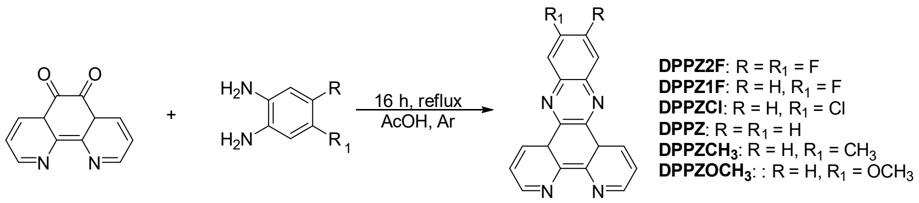

Scheme S1. Synthesis of di-pyridyl-phenazil-disubstituted derivatives.

DPPZ2F[14](purple solid, 88 %) ^{1}H NMR (300 MHz, $CDCl_3$) δ 9.64 (d, 1H), 9.34 (bs, 1H), 8.11 (t, 1H), 7.85 (dd, 1H).

DPPZ1F (violet solid, 98 %) ^{1}H NMR (300 MHz, $CDCl_3$) δ 9.63-9.59 (dt, 2H), 9.9-9.27 (dt, 2H), 8.38-8.35 (dd, 1H), 7.98-7.95 (dd, 1H), 7.82-7.78 (m, 2H), 7.75-7.70 (m, 1H).

DPPZCl (rose solid, 97 %) ^{1}H NMR (300 MHz, $CDCl_3$) δ 9.70 (d, 2H), 9.41 (bs, 2H), 8.39 (d, 1H), 8.33 (d, 1H), 7.92-7.86 (m, 3H).

DPPZ (rose solid, 94 %) ^{1}H NMR (300 MHz, $CDCl_3$) δ 9.65 (d, 1H), 9.27 (d, 1H), 8.35 (q, 1H), 7.93 (q, 1H), 7.81 (m, 1H).

DPPZCH3 (brown solid, 98 %) ^{1}H NMR (300 MHz, $CDCl_3$) δ 9.57 (d, 2H), 9.25 (d, 2H), 8.16 (d, 1H), 8.04 (s, 1H), 7.78-7.70 (m, 3H), 2.66 (s, 3H).

DPPZOCH3 (dark green solid, 97 %) ^{1}H NMR (300 MHz, $CDCl_3$) δ 9.63 (t, 2H), 9.31 (bs, 2H), 8.21 (d, 1H), 7.83 (bs, 2H), 7.61-7.56 (m, 2H), 4.09 (s, 3H).

Synthesis of alkylate phenanthroline and DPPZ derivatives.

In a flame dried and argon-purged round-bottomed flask equipped with a magnetic stir bar 1,10-phenantroline (150 mg, 0.83 mmol) or 1,10-phenantroline-5,6-dione (150 mg, 0.71 mmol) or the desired di-pyridyl-phenazil-disubstituted derivatives (200 mg) was put in 1,2-dibromoethane (4 mL). The reaction mixture was left under stirring for 3 days at 140°C (*Scheme S2*). Afterward acetone and *n*-hexane were added and the product was precipitated. It was filtered, washed with acetone and *n*-hexane and dried under vacuum. The products were obtained with a high yield as powders.

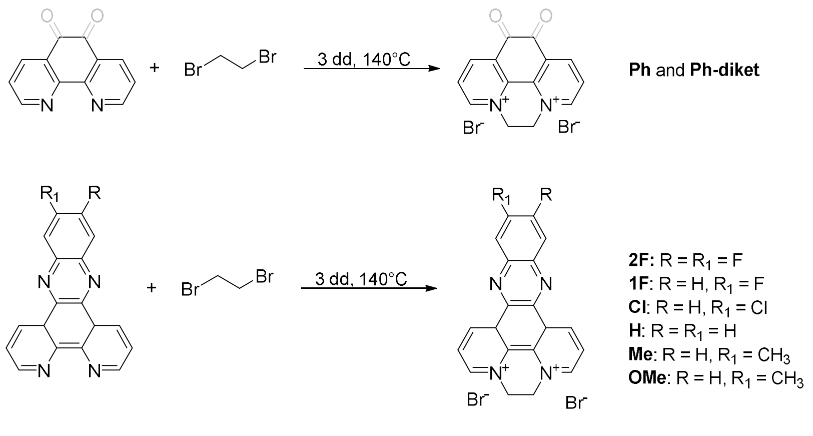

Scheme S2. Synthesis of alkylate phenanthroline and DPPZ derivatives.

2F (98 %) ^{1}H NMR (300 MHz, D_2O) δ 10.65 (d, 1H), 9.76 (d, 1H), 8.90 (dd, 1H), 8.50 (t, 1H), 5.78 (s, 2H).

1F (70 %) ^{1}H NMR (300 MHz, D_2O) δ 10.53 (d, 2H), 9.64 (t, 2H), 8.75 (dt, 2H), 8.61 (dd, 1H), 8.20 (dd, 1H), 8.05 (dt, 1H), 5.70 (s, 4H).

Cl (75 %) ^{1}H NMR (300 MHz, D_2O) δ 10.58 (d, 2H), 9.71 (d, 2H), 8.85-8.80 (dd, 2H), 8.63 (s, 1H), 8.56 (d, 1H), 8.20 (d, 1H), 5.77 (s, 4H).

H (90 %) ^{1}H NMR (300 MHz, D_2O) δ 10.63 (d, 1H), 9.73 (d, 1H), 8.83 (dd, 1H), 8.62 (m, 1H), 8.30 (m, 1H), 5.80 (s, 2H).

Me (75 %) ^{1}H NMR (300 MHz, D_2O) δ 10.54 (d, 2H), 9.72 (d, 2H). 8.82 (t, 2H), 8.40 (d, 1H), 8.29 (s, 1H), 8.10 (d, 1H), 5.80 (s, 4H), 2.75 (s, 3H).

OMe (97 %) ^{1}H NMR (300 MHz, D_2O) δ 10.44 (m, 2H), 9.58 (t, 2H), 8.69 (s, 2H), 8.28 (t, 1H), 7.73-7.49 (m, 2H), 5.67 (s, 4H), 4.05 (s, 3H).

Ph (98 %) ^{1}H NMR (300 MHz, D_2O) δ 9.69 (d, 1H), 9.58 (d, 1H), 8.76 (s, 1H), 8.67 (dd, 1H), 5.76 (s, 2H).

^{1}H NMR spectra of compounds.

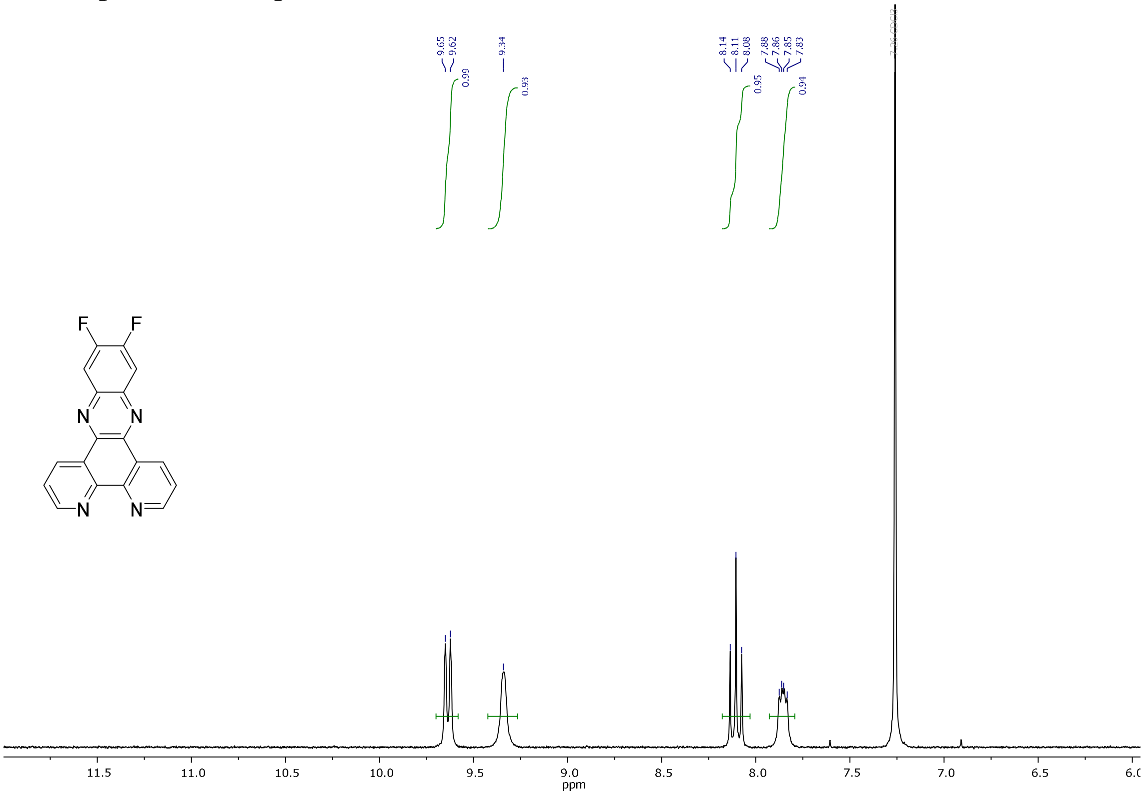

Figure S31. ^{1}H NMR ($CDCl_3$, 298 K) of **DPPZ2F**.

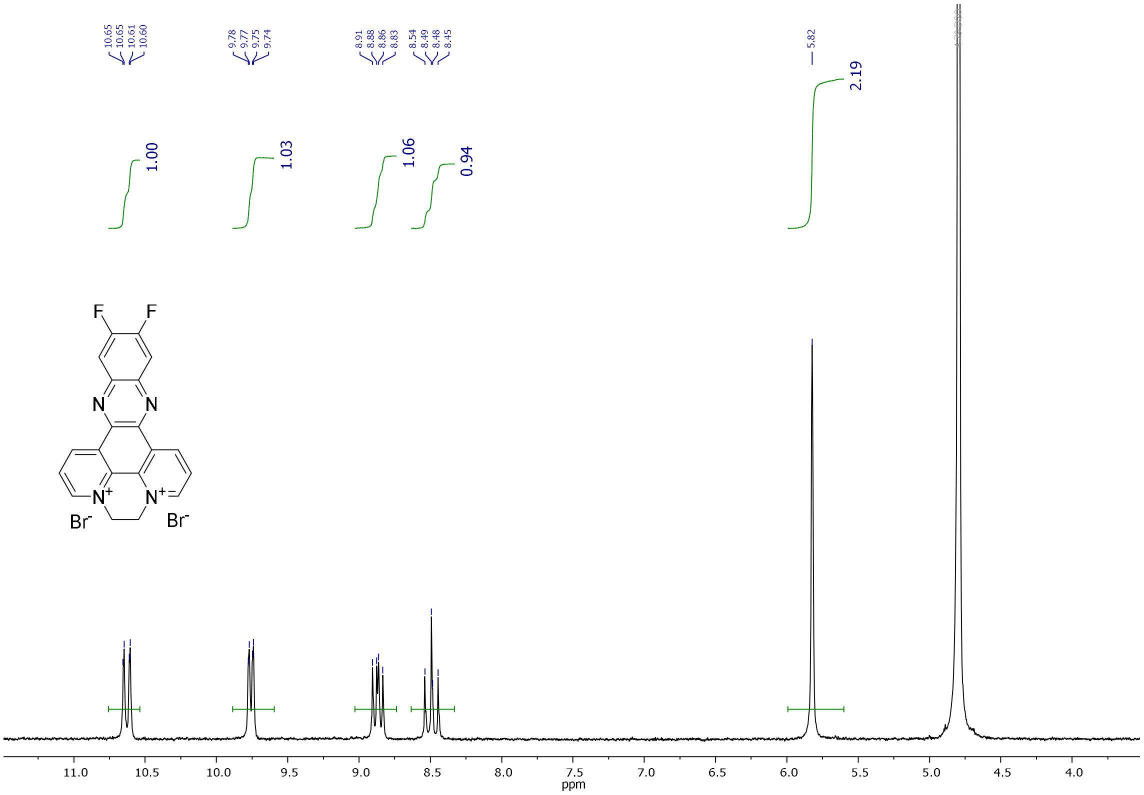

Figure S32. ^{1}H NMR (D_2O, 298 K) of **2F.**

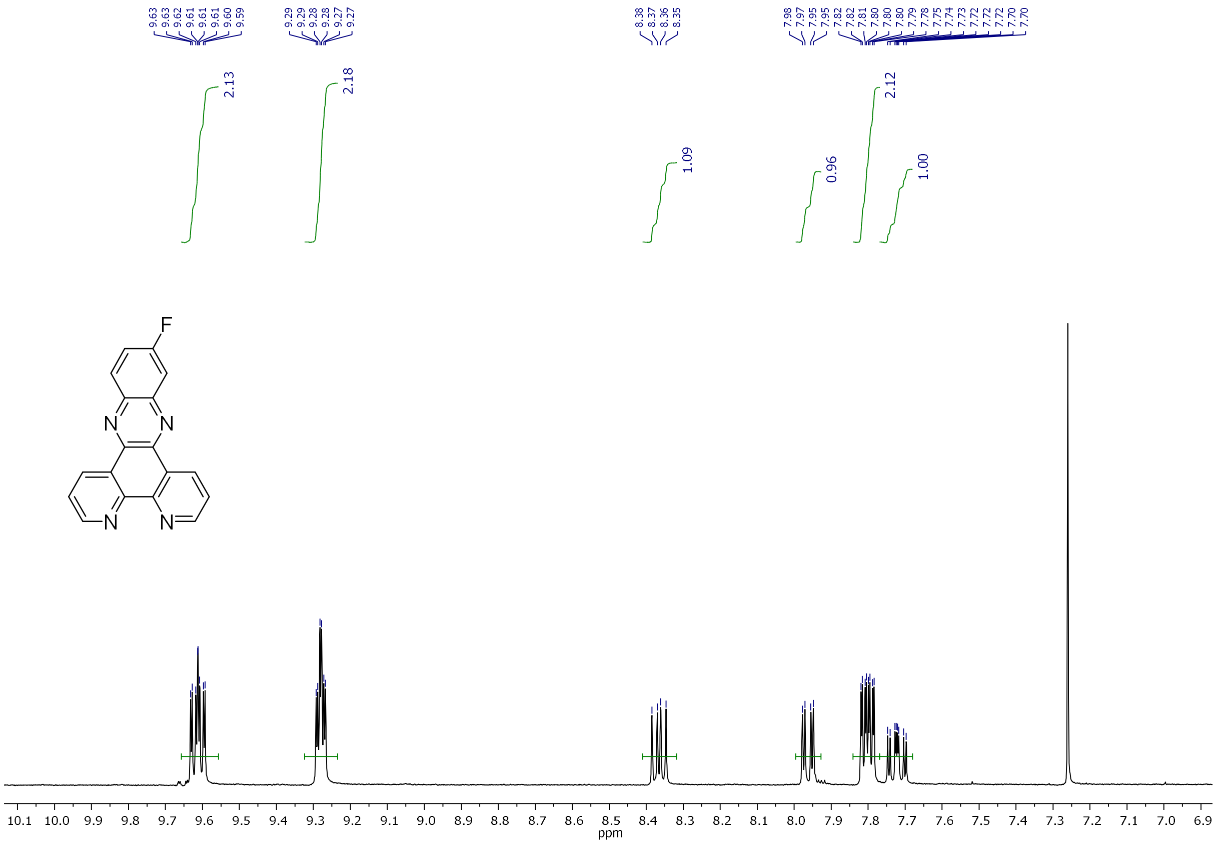

Figure S33. ^{1}H NMR ($CDCl_3$, 298 K) of **DPPZ1F**.

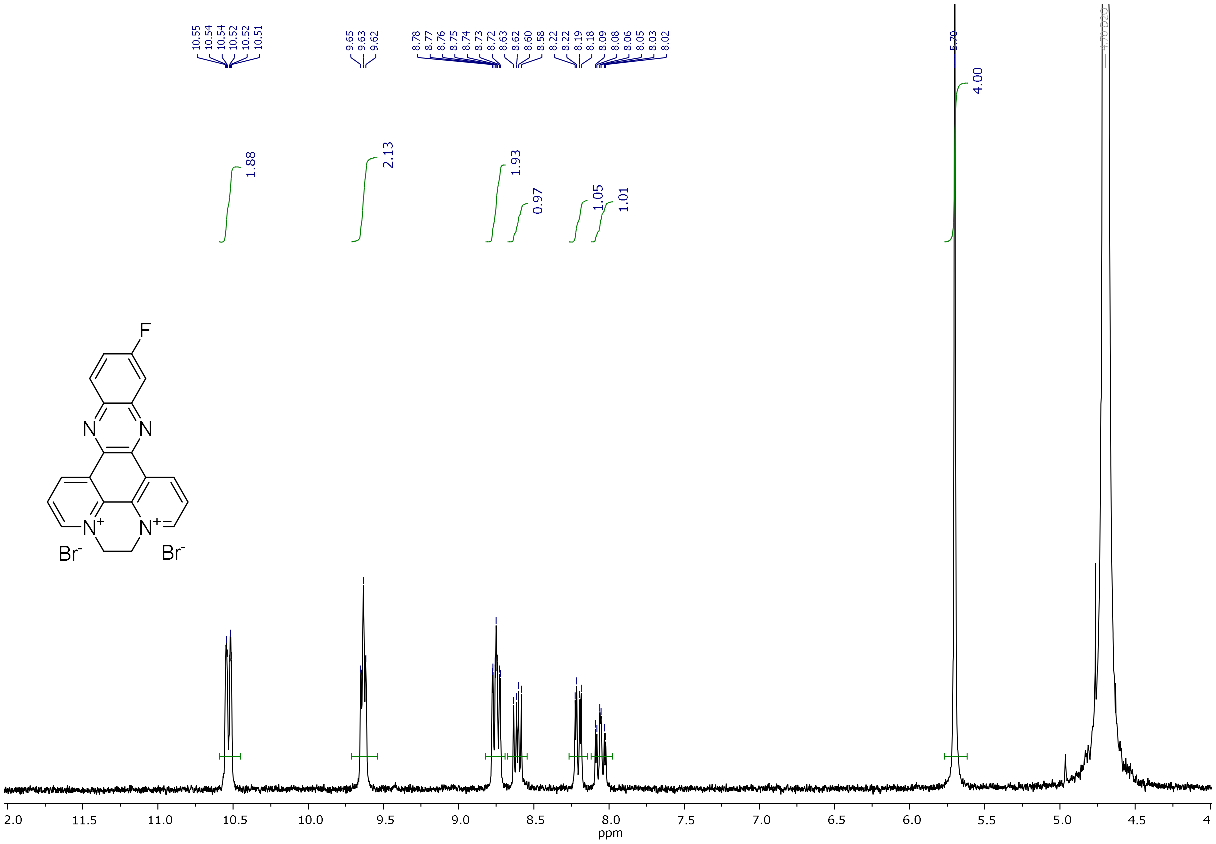

Figure S34. ^{1}H NMR (D_2O, 298 K) of **1F**.

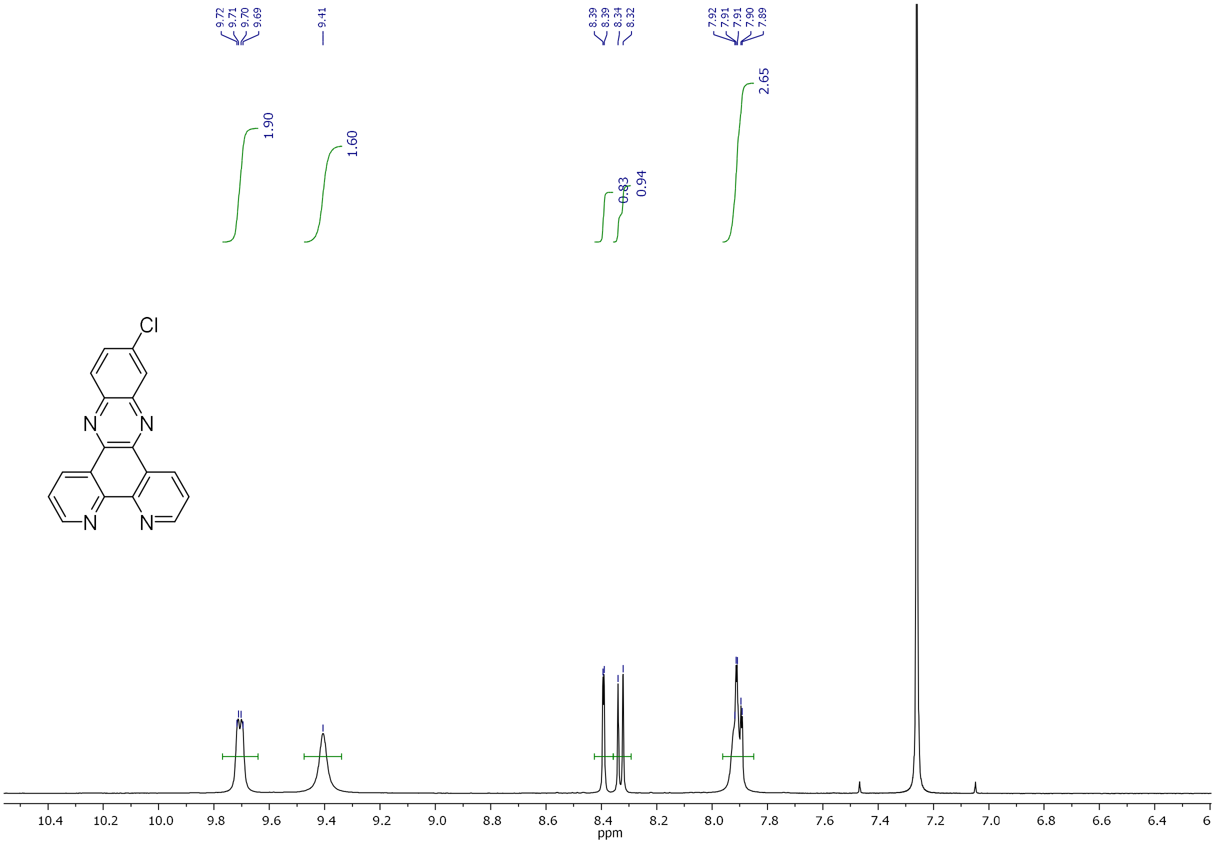

Figure S35. ^{1}H NMR ($CDCl_3$, 298 K) of **DPPZCl**.

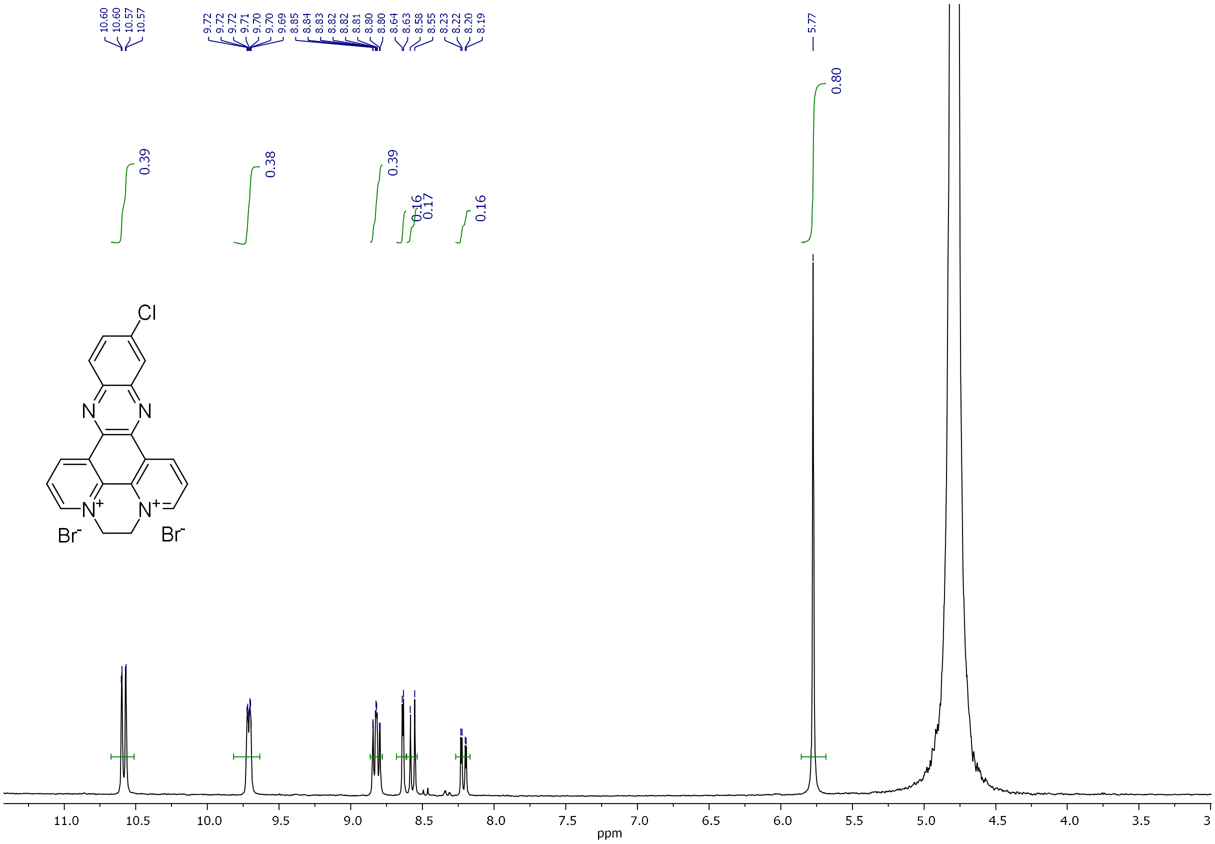

Figure S36. ^{1}H NMR (D_2O, 298 K) of **Cl**.

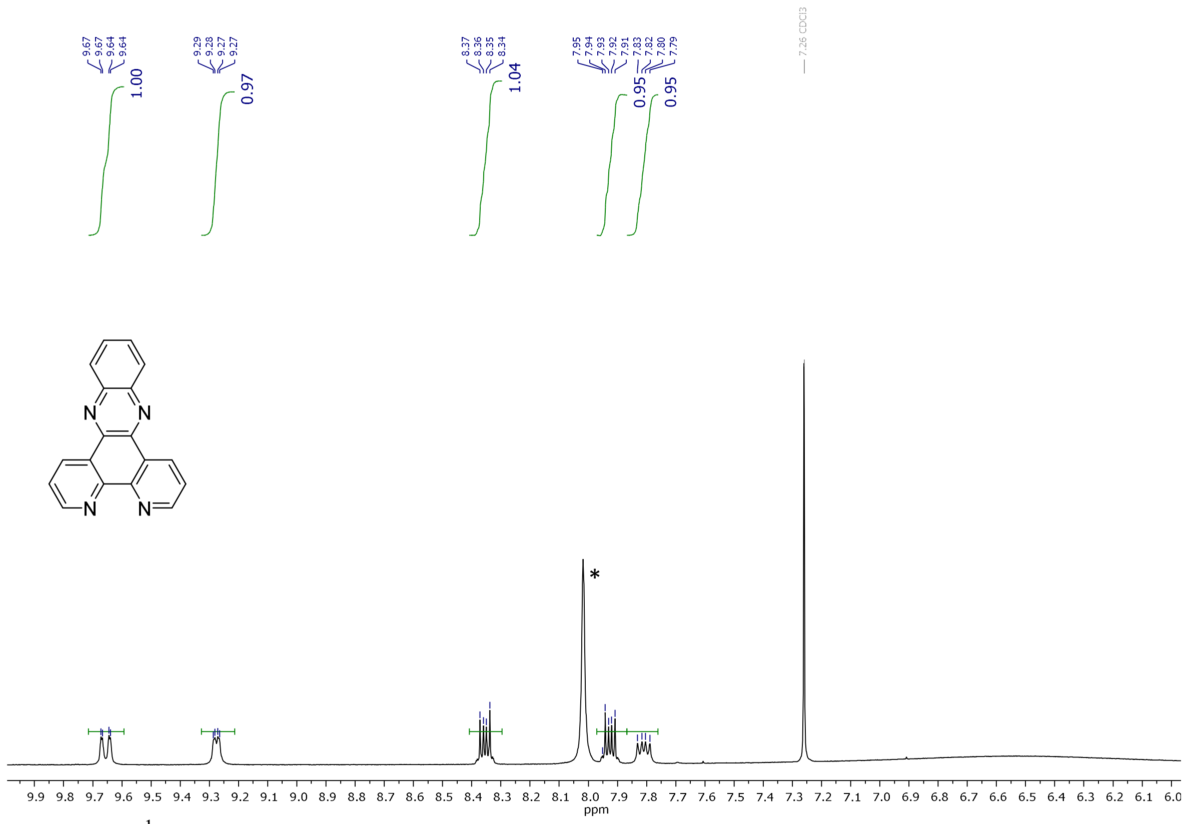

Figure S37. ^{1}H NMR ($CDCl_3$, 298 K) of **DPPZ**.

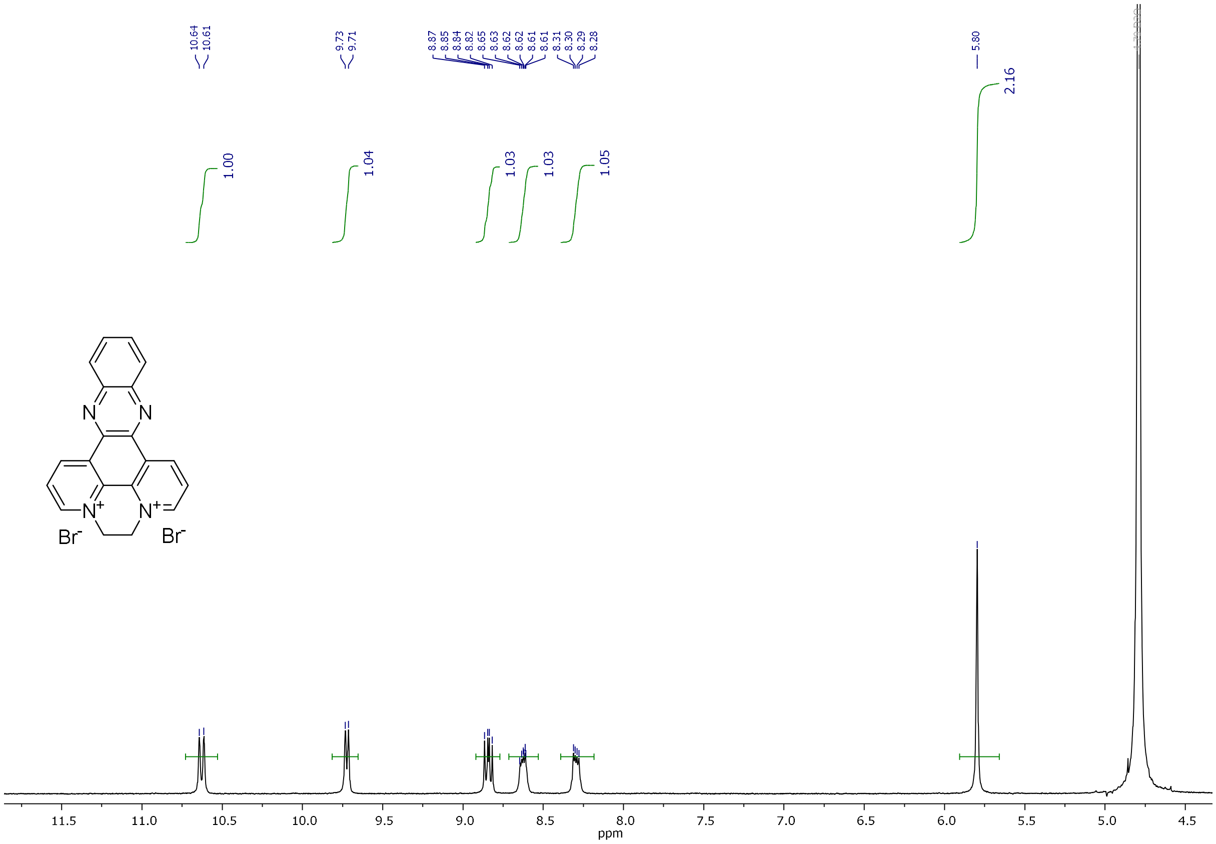

Figure S38. ^{1}H NMR (D_2O, 298 K) of **H**.

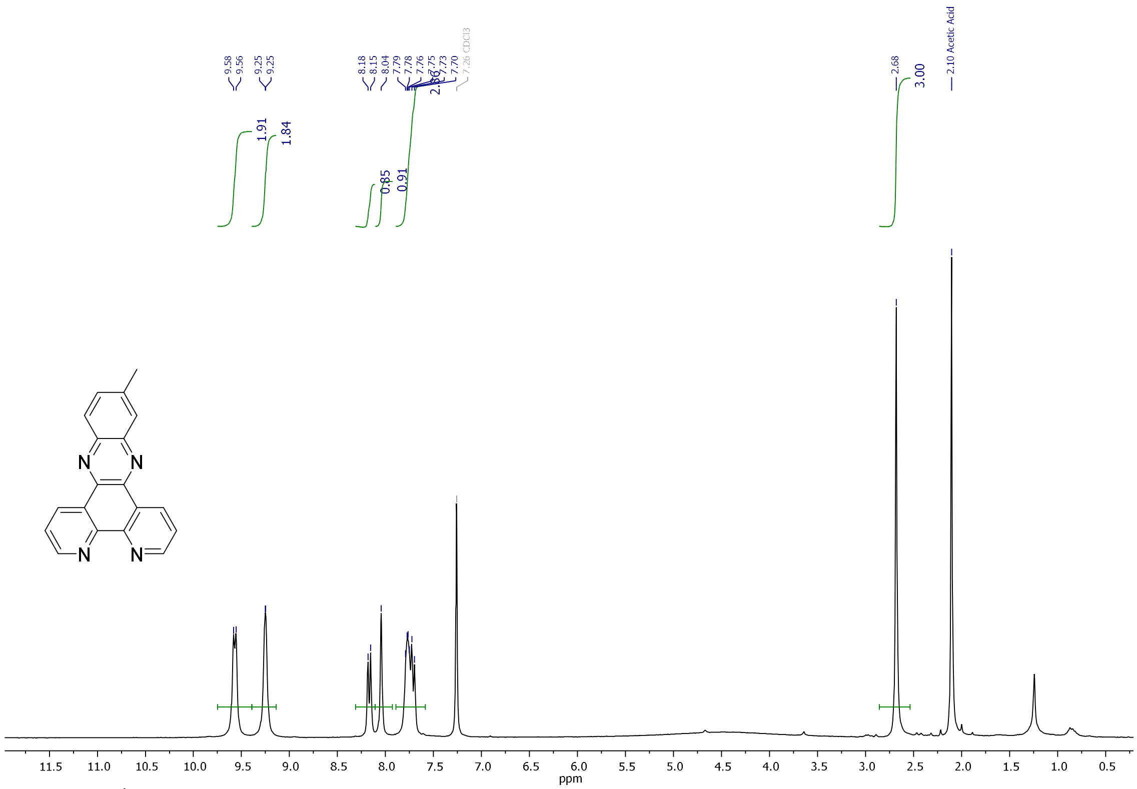

Figure S39. ^{1}H NMR ($CDCl_3$, 298 K) of **DPPZCH$_3$**.

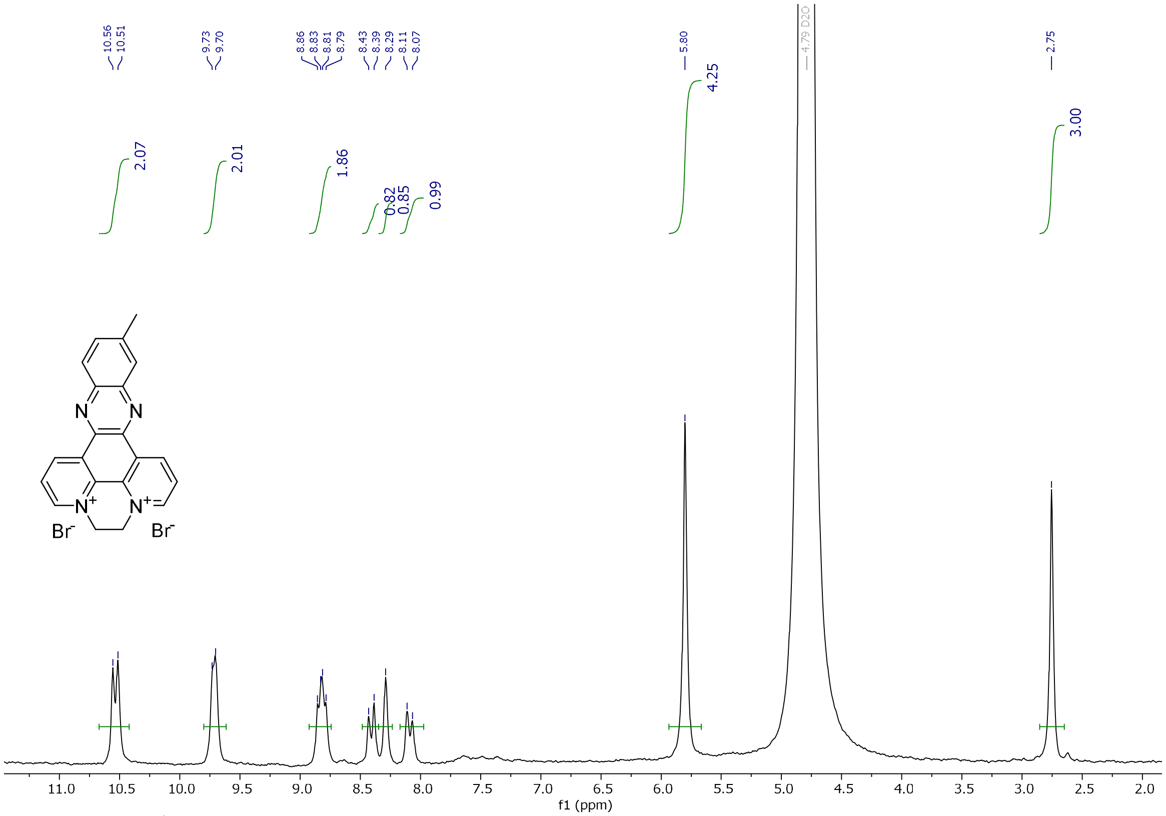

Figure S40. ^{1}H NMR (D_2O, 298 K) of **Me**.

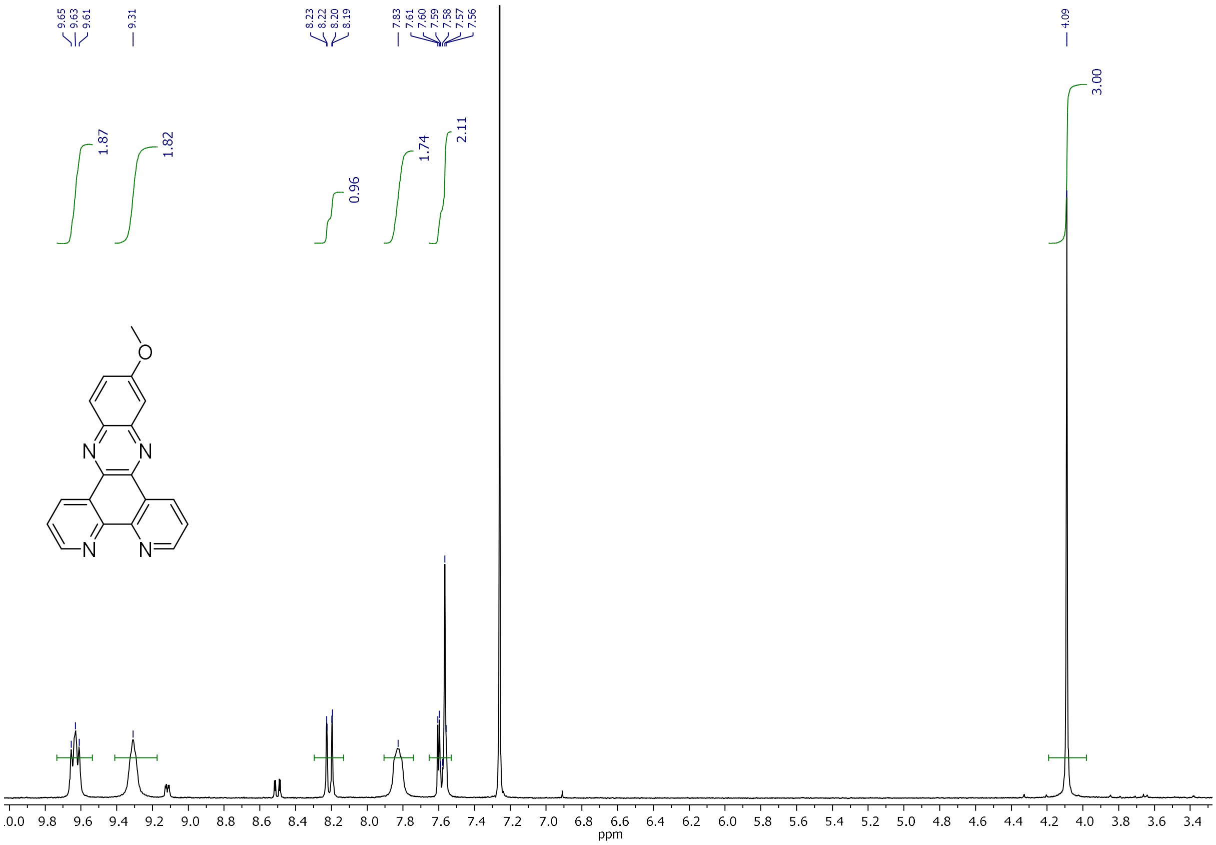

Figure S41. ^{1}H NMR ($CDCl_3$, 298 K) of **DPPZOCH₃**.

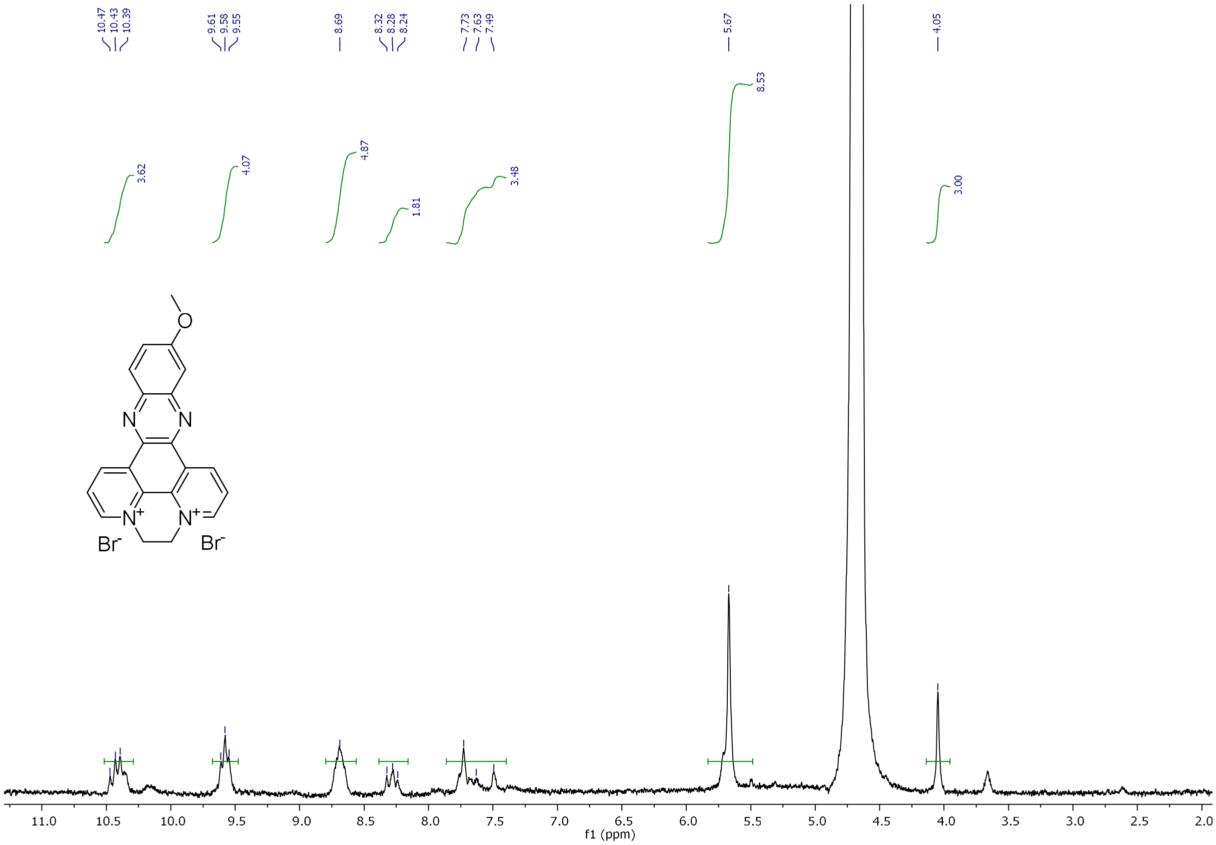

Figure S42. ^{1}H NMR (D_2O, 298 K) of **OMe**.

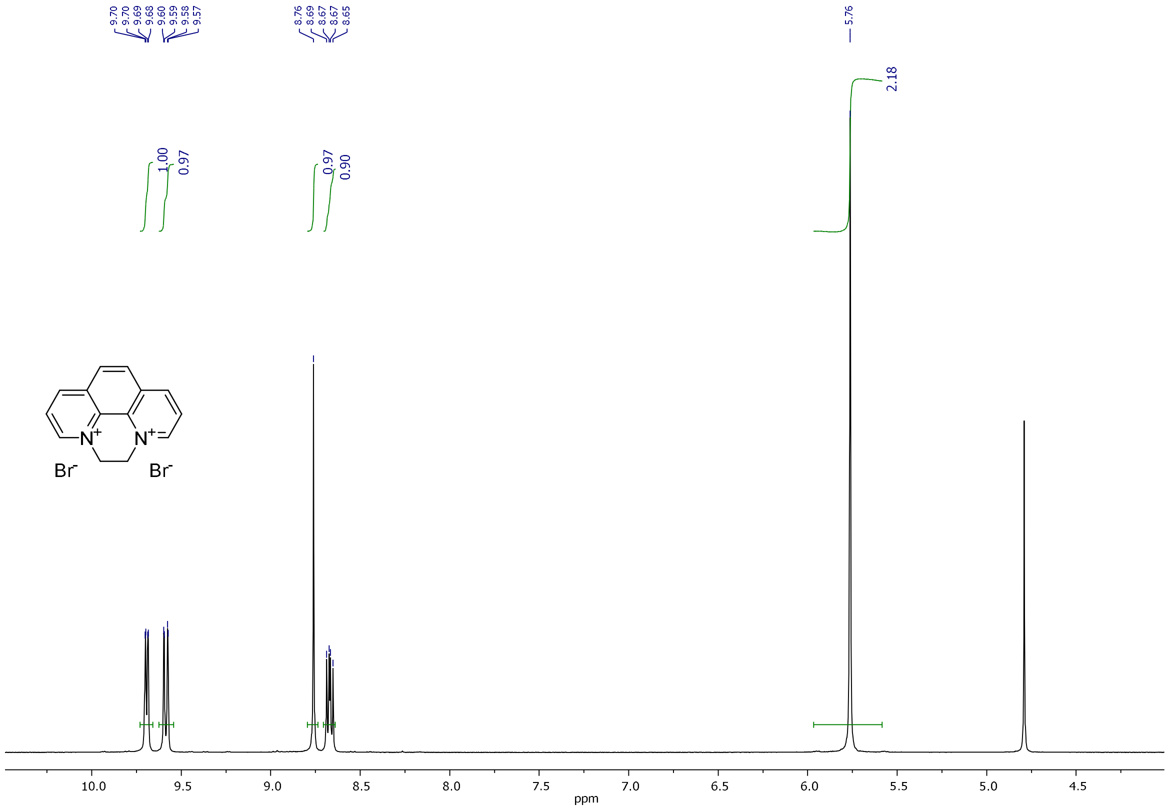

Figure S43. ^{1}H NMR (D_2O, 298 K) of **Ph**.

References

(1) Han, L.; Tian, B.; Gao, X.; Zhong, Y.; Wang, S.; Song, S.; Wang, Z.; Zhang, Y.; Kuang, Y.; Sun, X. Copper Nanowire with Enriched High-index Facets for Highly Selective CO $_2$ Reduction. *SmartMat* **2022**, *3* (1), 142–150. https://doi.org/10.1002/smm2.1082.

(2) Han, Z.; Han, D.; Chen, Z.; Gao, J.; Jiang, G.; Wang, X.; Lyu, S.; Guo, Y.; Geng, C.; Yin, L.; Weng, Z.; Yang, Q.-H. Steering Surface Reconstruction of Copper with Electrolyte Additives for CO2 Electroreduction. *Nat Commun* **2022**, *13* (1), 3158. https://doi.org/10.1038/s41467-022-30819-1.

(3) Lin, Y.; Wang, T.; Zhang, L.; Zhang, G.; Li, L.; Chang, Q.; Pang, Z.; Gao, H.; Huang, K.; Zhang, P.; Zhao, Z.-J.; Pei, C.; Gong, J. Tunable CO2 Electroreduction to Ethanol and Ethylene with Controllable Interfacial Wettability. *Nat Commun* **2023**, *14* (1), 3575. https://doi.org/10.1038/s41467-023-39351-2.

(4) Zhong, D.; Zhao, Z.; Zhao, Q.; Cheng, D.; Liu, B.; Zhang, G.; Deng, W.; Dong, H.; Zhang, L.; Li, J.; Li, J.; Gong, J. Coupling of Cu(100) and (110) Facets Promotes Carbon Dioxide Conversion to Hydrocarbons and Alcohols. *Angew Chem Int Ed* **2021**, *60* (9), 4879–4885. https://doi.org/10.1002/anie.202015159.

(5) Asperti, S.; Hendrikx, R.; Gonzalez-Garcia, Y.; Kortlever, R. Benchmarking the Electrochemical CO $_2$ Reduction on Polycrystalline Copper Foils: The Importance of Microstructure Versus Applied Potential. *ChemCatChem* **2022**, *14* (21), e202200540. https://doi.org/10.1002/cctc.202200540.

(6) Thevenon, A.; Rosas-Hernández, A.; Peters, J. C.; Agapie, T. In-Situ Nanostructuring and Stabilization of Polycrystalline Copper by an Organic Salt Additive Promotes Electrocatalytic

CO_2 Reduction to Ethylene. *Angewandte Chemie* **2019**, *131* (47), 17108–17114. https://doi.org/10.1002/ange.201907935.
(7) Cao, Y.; Chen, Z.; Li, P.; Ozden, A.; Ou, P.; Ni, W.; Abed, J.; Shirzadi, E.; Zhang, J.; Sinton, D.; Ge, J.; Sargent, E. H. Surface Hydroxide Promotes CO2 Electrolysis to Ethylene in Acidic Conditions. *Nat Commun* **2023**, *14* (1), 2387. https://doi.org/10.1038/s41467-023-37898-8.
(8) Li, J.; Xiong, H.; Liu, X.; Wu, D.; Su, D.; Xu, B.; Lu, Q. Weak CO Binding Sites Induced by Cu–Ag Interfaces Promote CO Electroreduction to Multi-Carbon Liquid Products. *Nat Commun* **2023**, *14* (1), 698. https://doi.org/10.1038/s41467-023-36411-5.
(9) Peng, Y.; Zhan, C.; Jeon, H. S.; Frandsen, W.; Cuenya, B. R.; Kley, C. S. Organic Thin Films Enable Retaining the Oxidation State of Copper Catalysts during CO_2 Electroreduction. *ACS Appl. Mater. Interfaces* **2024**, acsami.3c14554. https://doi.org/10.1021/acsami.3c14554.
(10) Kim, J.; Lee, T.; Jung, H. D.; Kim, M.; Eo, J.; Kang, B.; Jung, H.; Park, J.; Bae, D.; Lee, Y.; Park, S.; Kim, W.; Back, S.; Lee, Y.; Nam, D.-H. Vitamin C-Induced CO2 Capture Enables High-Rate Ethylene Production in CO2 Electroreduction. *Nat Commun* **2024**, *15* (1), 192. https://doi.org/10.1038/s41467-023-44586-0.
(11) Nie, W.; Heim, G. P.; Watkins, N. B.; Agapie, T.; Peters, J. C. Organic Additive-derived Films on Cu Electrodes Promote Electrochemical CO_2 Reduction to C_{2+} Products Under Strongly Acidic Conditions. *Angew Chem Int Ed* **2023**, *62* (12), e202216102. https://doi.org/10.1002/anie.202216102.
(12) Lai, Y.; Watkins, N. B.; Rosas-Hernández, A.; Thevenon, A.; Heim, G. P.; Zhou, L.; Wu, Y.; Peters, J. C.; Gregoire, J. M.; Agapie, T. Breaking Scaling Relationships in CO_2 Reduction on Copper Alloys with Organic Additives. *ACS Cent. Sci.* **2021**, *7* (10), 1756–1762. https://doi.org/10.1021/acscentsci.1c00860.
(13) Han, Z.; Kortlever, R.; Chen, H.-Y.; Peters, J. C.; Agapie, T. CO_2 Reduction Selective for $C_{\geq 2}$ Products on Polycrystalline Copper with N-Substituted Pyridinium Additives. *ACS Cent. Sci.* **2017**, *3* (8), 853–859. https://doi.org/10.1021/acscentsci.7b00180.
(14) S. Roy, E. Colombo, R. Vinck, C. Mari, R. Rubbiani, M. Patra and Gilles Gasser, *ChemBioChem*, **2020**, 21, 2966-2973.
